# Enhancing global preparedness during an ongoing pandemic from partial and noisy data

**DOI:** 10.1093/pnasnexus/pgad192

**Published:** 2023-06-07

**Authors:** Pascal P Klamser, Valeria d’Andrea, Francesco Di Lauro, Adrian Zachariae, Sebastiano Bontorin, Antonello Di Nardo, Matthew Hall, Benjamin F Maier, Luca Ferretti, Dirk Brockmann, Manlio De Domenico

**Affiliations:** Robert Koch-Institute, Nordufer 20, 13353 Berlin, Germany; Department of Biology, Institute for Theoretical Biology, Humboldt-University of Berlin, Philippstr. 13, 10115 Berlin, Germany; Fondazione Bruno Kessler, Via Sommarive 18, 38123, Povo (TN), Italy; Big Data Institute, University of Oxford, Old Road Campus, OX3 7LF Oxford, UK; Robert Koch-Institute, Nordufer 20, 13353 Berlin, Germany; Department of Biology, Institute for Theoretical Biology, Humboldt-University of Berlin, Philippstr. 13, 10115 Berlin, Germany; Fondazione Bruno Kessler, Via Sommarive 18, 38123, Povo (TN), Italy; Department of Physics, University of Trento, Via Sommarive 14, 38123 Povo (TN), Italy; The Pirbright Institute, Ash Road, GU24 0NF Surrey, UK; Big Data Institute, University of Oxford, Old Road Campus, OX3 7LF Oxford, UK; Robert Koch-Institute, Nordufer 20, 13353 Berlin, Germany; Department of Biology, Institute for Theoretical Biology, Humboldt-University of Berlin, Philippstr. 13, 10115 Berlin, Germany; Big Data Institute, University of Oxford, Old Road Campus, OX3 7LF Oxford, UK; Robert Koch-Institute, Nordufer 20, 13353 Berlin, Germany; Department of Biology, Institute for Theoretical Biology, Humboldt-University of Berlin, Philippstr. 13, 10115 Berlin, Germany; Department of Physics and Astronomy, G. Galilei, University of Padua, Via Francesco Marzolo 8, 35131 Padua, Italy; Padua Center for Network Medicine, University of Padua, Via Francesco Marzolo 8, 35131 Padua, Italy

**Keywords:** SARS-CoV-2/COVID-19, phylogeny, variant of concern, air-transportaion network, complex system

## Abstract

As the coronavirus disease 2019 spread globally, emerging variants such as B.1.1.529 quickly became dominant worldwide. Sustained community transmission favors the proliferation of mutated sub-lineages with pandemic potential, due to cross-national mobility flows, which are responsible for consecutive cases surge worldwide. We show that, in the early stages of an emerging variant, integrating data from national genomic surveillance and global human mobility with large-scale epidemic modeling allows to quantify its pandemic potential, providing quantifiable indicators for pro-active policy interventions. We validate our framework on worldwide spreading variants and gain insights about the pandemic potential of BA.5, BA.2.75, and other sub- and lineages. We combine the different sources of information in a simple estimate of the pandemic delay and show that only in combination, the pandemic potentials of the lineages are correctly assessed relative to each other. Compared to a country-level epidemic intelligence, our scalable integrated approach, that is pandemic intelligence, permits to enhance global preparedness to contrast the pandemic of respiratory pathogens such as SARS-CoV-2.

**Significance Statement**

The SARS-CoV-2 pandemic claimed during the last 2 years millions of deaths despite the mitigating effects of nonpharmaceutical interventions and model predictions that prepared decision makers. In fact, predictions became obsolete with emergent variants higher immune-escape and/or increased infectiousness. Even if their epidemic characteristics were known, their distant origin introduced additional uncertainty. We combine phylogenetic information from a small number of the first sequenced probes with epidemic- and human mobility information to provide country-specific epidemic projections and a simple estimate of the pandemic delay that allows an inter-lineage comparison. This global approach enables countries, especially those with low sequencing rate, to estimate when current mitigation measures need adaptation to stay efficient.

## Introduction

The coronavirus disease (COVID-19) outbreak, caused by the SARS-CoV-2 virus and first detected in China in early 2020, likely originated from the Huanan seafood wholesale market in Wuhan (1) and continues to spread worldwide. It has forced national governments to pursue country-level elimination strategies (4, 2, 3) or mitigation policies relying on both nonpharmaceutical interventions (NPI)—for example, physical distancing, wearing masks, hand hygiene, limit large gathering of people, curfews and, in the worst cases, lockdowns (5)—and pharmaceutical ones, such as massive vaccination campaigns and antiviral therapies (8, 6, 7). Early strict interventions have been shown to be more effective than longer moderate ones in containing national outbreaks in curbing epidemic growth (9), for similar intermediate distress and infringement on individual freedom (10).

In contrast to policy during the early stages of the pandemic, when pharmaceutical interventions were not yet available, most current national efforts to control the virus rely on reactive strategies which alternate enhancement and lifting of NPIs, with the ultimate goal of prevention, or reduction, of pressure on national health systems. To achieve successful containment, such reactive strategies require high capacity for testing and sequencing to continuously monitor the potential emergence of novel viral strains of SARS-CoV-2, whose mutations might be responsible for more severe and/or more transmissible variants with pandemic potential (11). We define pandemic potential as the ability of a variant to escape population immunity acquired by vaccination or previous infections and to cause quickly spreading infections worldwide. Note, that the acquired immunity may still confer protection against severe disease and thus the definition does not include the variant’s disease severity, which cannot be estimated from limited, early sequencing data alone. That means a variant with high pandemic potential does not strictly require the strengthening of mitigation measures but suggests to thoroughly re-evaluate them due to an expected fast global spread.

Although the emergence of within-host variants with immune escape is likely to be relatively rare (12), sustained community transmission might favor it. When a new variant emerges, it is crucial for policy and decision-making to characterize novel mutations (15, 13, 14), estimate the growth advantage of the new variant with respect to the existing ones (16), and quantify the effectiveness of currently available vaccines (17, 18). Consequently, any delay in identifying an emerging variant and in determining its key epidemiological parameters introduces uncertainties in the timeline of community transmissions and imported cases which limit, if not completely prevent, effective mitigation responses to take place, similarly to the cryptic transmission of the wild type SARS-CoV-2 which led to the first COVID-19 wave (19). Combined with limited testing capacity, porous travel screening (20)—at national and, overall, cross-national levels, where international travel play a significant role to amplify the pandemic potential (21, 22, 19)—and lifting of national NPI, the same delays might seriously hinder the timely detection of an emerging variant. The COVID-19 pandemic has been characterized by the regular emergence of such variants (23). Three important questions arise during the early stages of such a variant, at which point data is missing and noisy: (i) can we reconstruct its geographical origin? (ii) can we estimate how long it has been spreading undetected in that location? and (iii) can we quantify the risk of importation to other locations?

This work answers these questions by three major contributions. First, we derive a protocol integrating phylogenetic, epidemiological, and behavioral analyses within a framework for data-driven and model-informed pandemic intelligence. Second, we demonstrate that with limited, early and noisy sequencing data, it is possible to quantify the pandemic potential of an emerging variant and predict the dynamics of subsequent national outbreaks with satisfactory precision. Finally, we propose a simple combination of the different sources of information to qualitatively compare the lineages according to their pandemic delay and find that only the combined measure can reproduce the observed differences.

## Results

*Blueprint for a pandemic intelligence framework*. Reliably quantifying the pandemic potential of an emerging variant requires data, and acquiring data requires time. Between the time $t_{0}$ of the first undetected case and the time $t_{1}$ of the first reported case and its subsequent lineage designation at time $t_{2}$, an emerging variant can silently spread within its country of origin and beyond. For example, let us consider the B.1.1.529 lineage of the Omicron variant (also known as BA.1). This was first reported by genomic surveillance teams in South Africa and Botswana on November 25th 2021. Priority actions have been established by the World Health Organization (WHO) for member states on November 26th, with designation as a variant of concern (VOC) (24) required to raise the level of international alert ($t_{3}$). By December 16th 2021, there were several reports of an estimated reduction in both vaccine effectiveness against infection and severe disease (28, 25, 27, 18, 26), together with characterizations of the epidemiology of the variant in South Africa (29), Denmark (30), and Norway (31). Early phylogenetic analysis placed $t_{0}$ during the third week of October 2021, about 1 month before $t_{1}$. Three weeks later it had been identified in 87 countries (29).

**Fig. 1.**
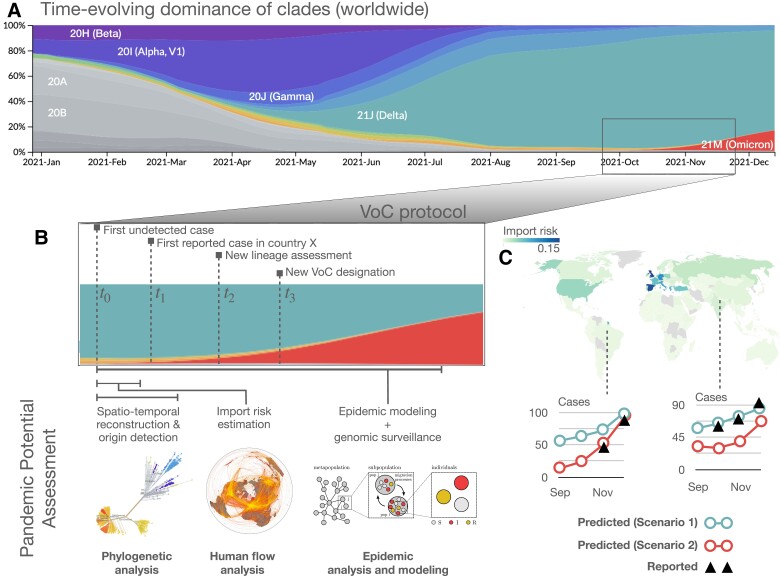
Schematic illustration of our pandemic intelligence workflow. A) Evolutionary dynamics of SARS-CoV-2 variants, coded by colour. The panel is obtained from nextstrain.org, based on GISAID data. B) For the B.1.1.529 lineage (or BA.1, *Omicron*, according to the WHO nomenclature), we identify four distinct time points in the process of characterizing the variant, from the time of the first undetected case to the designation as Variant of Concern. This illustrates how genomic surveillance data is used in combination with global human movement data and epidemic modeling to: (i) perform a spatiotemporal reconstruction of the patient zero to identify the country of origin of an emerging variant and estimate its epidemiological parameters and (ii) calculate the importation risk for all other countries worldwide. C) For a subset of about 50 countries worldwide (depending on sequencing data availability), we forecast the increase in the number of cases due to the consequent community transmission according to what-if scenarios, accounting for distinct levels of under-reporting. For a more mechanistic workflow scheme, see Supplementary Fig. S1.

Fig. 1 summarizes this timeline for B.1.1.529, while highlighting the main analytical steps required to define a self-consistent protocol to characterize the pandemic potential of an emerging variant (see Supplementary Fig. S1 for more mechanistic scheme). Fig. 1B illustrates how genomic surveillance data and epidemic modeling can be used to infer the spatio-temporal coordinates of the variant’s origin, thus providing information on $t_{0}$. This information is used to estimate the importation risk for all countries in the world due to cross-national human flows. Finally, imported cases are used as seeds for community transmission leading to country-level outbreaks, while accounting for the epidemiological parameters characterizing the new variant. Unavoidable uncertainties about $t_{0}$ and epidemiological parameters are propagated through the workflow. Plausible scenarios are presented, accounting for distinct levels of case under-reporting in each destination country (Fig. 1C).

In the following, we describe each step of the procedure, detailing our pandemic intelligence framework and the underlying modeling assumptions.

*Step 1: Reconstructing the origin of an emerging variant in space and time and its epidemic parameters*. For all SARS-CoV-2 sequences belonging to the B.1.1.7 (Alpha), B.1.617.2 (Delta), B.1.1.529, BA.2, BA.5, and BA.2.75 (Omicron) lineages from GISAID (32, 34, 33), we retain only those generated from cases reported during the early stage of the corresponding wave from the country of evolutionary origin, from 20 up to a total of 100 sequences per lineage. Where there are multiple candidate countries of origin, we estimate the outbreak country by a simple trait model. We then generate three alignments, comprised of respectively 20%, 50%, and 100% of the sequence set. These are subsequently cleaned by trimming the $5^{\prime }$ and $3^{\prime }$ untranslated regions and gap-only sites. Bayesian evolutionary reconstruction of the dated phylogenetic history (35) is used to obtain posterior distributions of the growth rate *t*, the parameters of the molecular clock, and the time of the most recent common ancestor (tMRCA). See Materials and Methods for details.

In this way, we obtain an estimate of $t_{0}$, the time of the first unreported case, as well as of other epidemic parameters such as the growth rate. From these, we estimate the effective reproduction number and generation interval. Alternatively, an emergent variant can be characterized by epidemic modeling: Indicating the number of infected individuals and number of deaths at time *t* by I(t) and D(t), respectively, we consider the time period during which there is co-circulation of an existing variant *v* and an emerging one ω. We approximate the epidemic evolution by


(1) $$\begin{array}{rl} I(t_{0}+\Delta t) & =I_{v}(t_{0}+\Delta t)+I_{\omega }(t_{0}+\Delta t)\\ & =I_{v}(t_{0})R_{v}(t_{0}{)}^{\Delta t/{\text{GI}}_{v}}+I_{w}(t_{0})R_{w}(t_{0}{)}^{\Delta t/{\text{GI}}_{\omega }}, \end{array}$$

where $I_{x}(t)$ is the number of infections due to variant *x* at time t, $R_{x}(t_{0})$ is the effective reproduction number at time $t_{0}$, and ${\text{GI}}_{x}$ is the generation interval. Similarly, the deaths due to the co-circulating variants are approximated by $D(t)=D_{v}(t)+D_{\omega }(t)$, with


(2) $$D_{x}(t_{0}+\Delta t+\tau_{x})=I_{x}(t_{0}+\Delta t)\times {\text{IFR}}_{x},\;x=v,w$$

where ${\text{IFR}}_{x}$ denotes the infection fatality rate of variant *x* and $\tau_{x}$ is the time lag between infection and death. To fit the unknown epidemiological parameters, that is the ones related to variant ω for which we obtain a joint probability distribution, we use an optimization procedure (see Materials and Methods section).

In the case of B.1.1.529, we obtain $t_{0}=29$ October 2021 (95% HPD: October 20–November 5) and a daily growth rate estimate of 0.566 (95% HPD: 0.117–1.035) from the phylogenetic analysis and $t_{0}=19$ October 2021 (95% CL: October 15–October 23) from epidemic modeling, with $R_{t}=2.56$ (95% CL: 2.16–3.19) and GI=7.36 (95% CL: 6.12–9.17). Our results are in good agreement with the literature, reporting $t_{0}=9$ October 2021 (95% HPD: September 30–October 20), exponential growth rate of 0.137 (95% HPD: 0.099–0.175) per day (29) and GI=6.84 days (95% credible intervals: 5.72–8.60) (36).

For further details, refer to Materials and Methods section and Supplementary Fig. S2.

*Step 2: Estimating the import risk of an emerging variant by country*. We use monthly seat capacities of flights between airports from the Official Airline Guide (37), encoding how many people could have traveled if all seats were occupied on flights from airport A to B in the month of the estimated $t_{0}$, that is differing between variants. We indicate the corresponding flow matrix by F, where entry $F_{ij}$ describes the maximal passenger flow to *i* from *j*. The traveling population in the catchment area of an airport is obtained by $N_{i}=F_{i}$, with $F_{i}=\sum_{j}F_{\;ji}$, that is, we assume that the population in the catchment area of the airport is equal to the airports outflow. The import risk is calculated as in (38): a random walker starts at the outbreak country and explores the flight network with $P_{ij}=F_{ij}/F_{j}$ as the transition probability to *i* from *j*. The walker has a node-specific probability to exit that is based on the effective distance graph (22) with the effective distance


(3) $$D_{ij}=d_{0}-\log\;(P_{ij}),$$

where $d_{0}$ is a constant that is added for each connecting flight. The import risk cumulates the walker’s exit behavior from all paths and estimates how likely it is that an infected individual from the emergent variant’s outbreak country reaches any airport worldwide (see Materials and Methods section, Supplementary Eq. S9). To work at country level, we aggregate the import risk of all airports of the outbreak country by computing the mean import risk weighted by the international outflux of each airport in the outbreak country. Note, that the effective distance $D_{ij}$ alone does not provide this information. We performed an extensive analysis to validate the estimated import risk against available data, such as the official arrival times as obtained from the WHO, for each emerging variant. We find considerable correlation between arrival time and import risk distance (Supplementary Eq. S13) for different variants (Alpha, Beta, Delta, Gamma) with a median of r=0.55 (range r∈[0.41,0.56]). This median is the largest compared with several alternative distance measures (see Supplementary Figs. S3–S5). Possible reasons for the medium correlation are reporting-uncertainties of the official arrival times (e.g. low genome sequencing rate) and the probabilistic nature of the infected passenger distribution. To ensure that it is not due to an incorrect estimated outbreak location, we identify likely candidates by recomputing the correlation for all countries as source (similar to (22)). For Beta, Gamma, and BA.1, the country declared by the WHO as the outbreak source has the greatest degree of correlation. For Delta and Alpha, the WHO candidate has the second and fifth best correlation respectively (see Supplementary Figs. S6 and S7). We extend the analysis to sub-lineages of Omicron and previously circulating variants of interest (VOIs) by estimating arrival times and outbreak countries from GISAID data. For 13 of 17 variants, the suspected outbreak location from GISAID has at least the third-largest correlation coefficient (of 183), and for all variants the GISAID candidate is at least on the 12th rank (see Supplementary Figs. S8–S10).

*Step 3: Modeling country-level epidemic spread of an emerging variant under distinct scenarios*. We use results from the previous step of the pipeline as inputs for an epidemic model in order to forecast the potential surge in cases due to an emerging variant in a target country. First, we estimate the daily number of infected people (seeds) traveling to the target country from the country where the VoC emerged (source country), based upon four elements: (i) results of our phylogenetic analysis, which inform both the growth rate and the time of emergence of the variant of concern, (ii) genomic surveillance in the source country, (iii) estimates of prevalence in the source country (incoporating under-reporting), and (iv) the import risk score of the target based on estimates from our analysis. Then, we produce short-term estimates of the daily incidence of the VoC in the target country by means of a Renewal process (40, 39, 41), in which we take into account both the introductions of seeds from the source country and the local epidemic dynamics caused by secondary cases. The renewal equation approach comes with three main advantages with respect to other models, such as SIR (42). In fact, (i) it does not require to include in the dynamics the immunological status of the population in the target country; (ii) the VoC dynamics can be considered as independent from the ones of the co-circulating VoCs, thus avoiding the need of estimating additional parameters for concurring spreading processes; (iii) the model explicitly includes the most relevant epidemiological observables, such as $R_{t}$, the serial interval distribution (43), and the immune escape of the VoC. For further details we refer to Materials and Methods section and Supplementary Figs. S11 and S12.

**Fig. 2.**
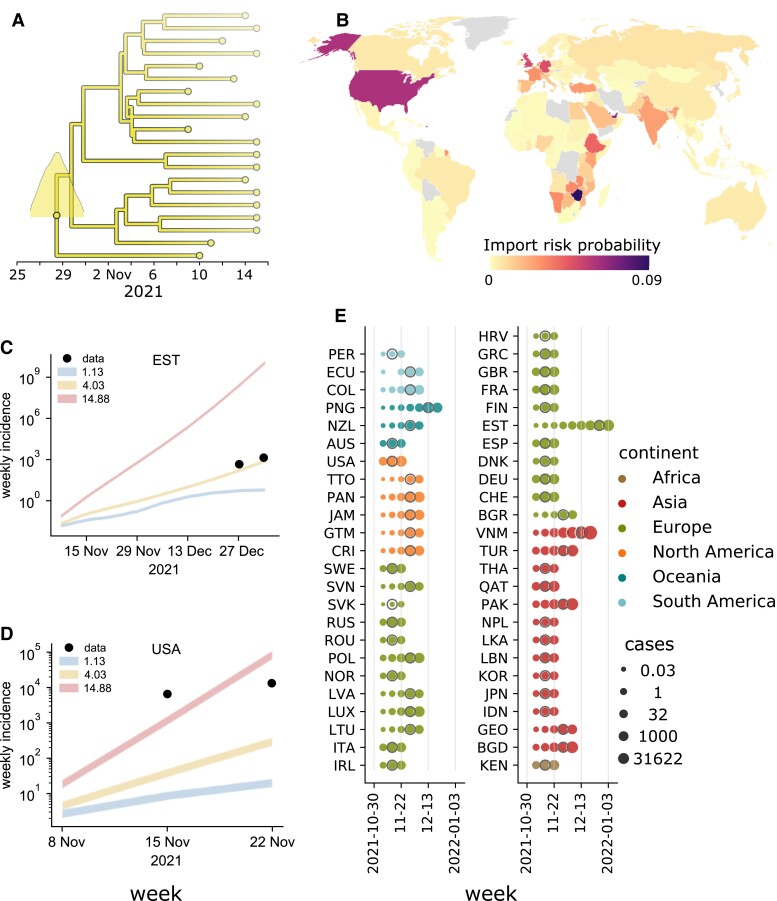
Quantifying the pandemic potential of the B.1.1.529 lineage. A) Phylogenetic reconstruction and estimation of the most recent common ancestor (MRCA), identified South Africa on October 28, 2021 (95% HPD: October 20–November 5) as the most likely MRCA. B) Import risk map: countries are colored by their probability to import infectious individuals carrying the B.1.1.529 (Omicron BA.1) lineage. C, D) Projected weekly incidence in Estonia and the United States obtained from epidemic modeling, under different $R_{t}$ scenarios indicated by colored lines, where the lowest line corresponds to the lowest *R_t_* scenario. Line thickness represents the range between the minimum and maximum assumed values of under-reporting in the source country (here South Africa). Points represent the observed incidence. E) Case counts simulated using the $R_{t}$ scenario that corresponds to the mean growth rate from the phylogenetic analysis. For each country, the date of the first reported case is indicated with a gray circle.

*Step 4: Assessing the pandemic potential of emerging variants*. In Fig. 2, we show the result of each step described above in determining the genomic and epidemiological parameters of the BA.1 lineage and, accordingly, quantify its pandemic potential. We refer the reader to Supplementary Figs. S13 and S14 for a more detailed analysis of errors in these estimates. Fig. 2A displays a time-resolved maximum clade credibility phylogeny of the lineage. Panel B is the map of import risk across the world. C, D) For two example countries, the simulated epidemic projections, plotted as weekly incidence. For each reproduction number, the shaded area represents the interval between the estimates derived using the minimum and maximum values of under-reporting in the source country. E) Model estimates of case counts in all considered countries are provided.

**Fig. 3.**
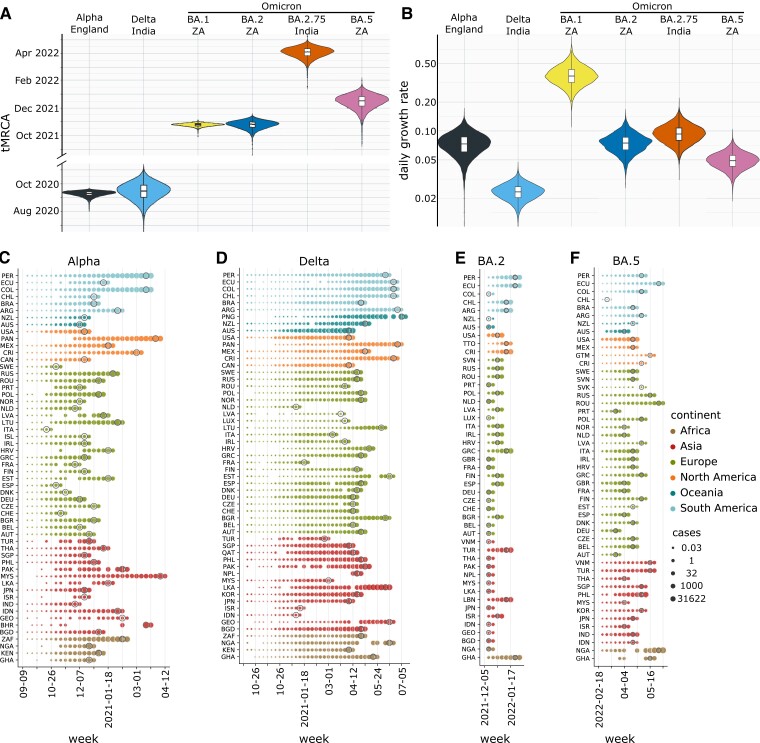
Pan-viral pandemic potential: comparing multiple lineages. A, B) tMRCA and growth rate estimates for Alpha, Delta, BA.1 (B.1.1.529), BA.2, BA.2.75, and BA.5 from phylogenetic analysis. C–F) Estimates of case numbers in all the considered countries for the same variants. For each lineage and country, the epidemic simulation starts at the time of infection $t_{0}$ of the first undetected case as identified using the phylogenetic analysis. The simulation stops at the third date at which sequences belonging to the considered lineages are greater than zero. Results are provided in logarithmic scale and dates at which the first case is reported are marked with gray circles.

Fig. 3 shows the results obtained for the SARS-CoV-2 lineages B.1.1.7 (Alpha), B.1.617.2 (Delta), B.1.1.529, BA.2, and BA.5 (Omicron). The date of the most recent common ancestors and the growth rate are shown, together with the temporal evolution of the number of expected cases around 50 countries (varies depending on available sequencing data; Alpha: 59, Delta: 55, BA.2: 51, BA.5: 49 countries). Point estimates of the mean and 95% HPD regions are further provided in Table 1.

**Table 1.** Phylogenetic estimates of the time of most recent common ancestor (tMRCA) and daily growth rate.

SARS-CoV-2 lineage	tMRCA [95% HPD]	Growth rate [95% HPD]
B.1.1.7 (Alpha)	Sep 10, 2020 [Aug 28–Sep 19, 2020]	0.091 [0.008–0.202]
B.1.617.2 (Delta)	Aug 25, 2020 [Jul 5–Oct 10, 2020]	0.020 [0.008–0.033]
B.1.1.529 (Omicron)	Oct 29, 2021 [Oct 20–Nov 5, 2021]	0.566 [0.117–1.035]
BA.2 (Omicron)	Oct 24, 2021 [Oct 4–Nov 9, 2021]	0.136 [0.046–0.262]
BA.5 (Omicron)	Jan 10, 2022 [Dec 19–Jan 29, 2022]	0.110 [0.051–0.177]
BA.2.75 (Omicron)	Apr 5, 2022 [Mar 11–Apr 23, 2022]	0.092 [0.037–0.162]

The values of the SARS-CoV-2 B.1.1.7 (Alpha), B.1.617.2 (Delta), B.1.1.529 (BA.1), BA.2, BA.5, and BA.2.75 (Omicron) lineages. Values are expressed as medians and 95% high posterior density intervals.

**Fig. 4.**
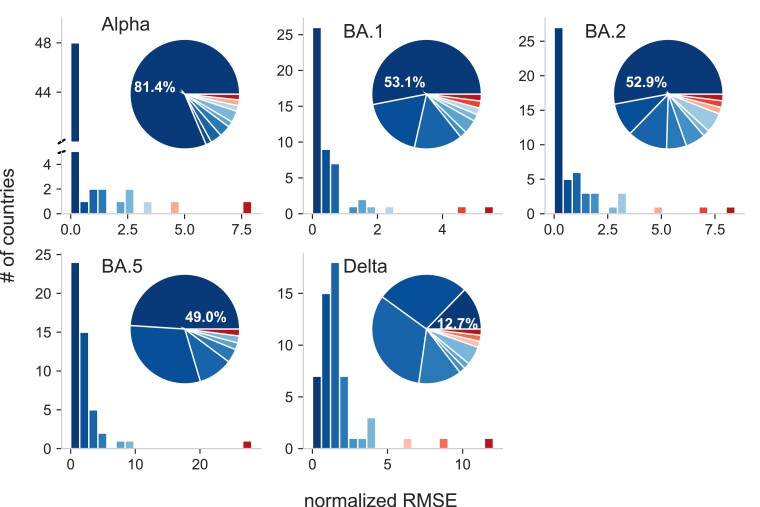
Pandemic intelligence workflow error estimation. Absolute (bars) and relative frequency (segments) of countries according to their normalized root-mean-square error (nRMSE) for the Alpha (B.1.1.7), Delta (B.1.617.2), BA.1 (B.1.1.529), BA.2, BA.5 (Omicron) lineages. The normalized RMSE is zero if the number of infected people evaluated from data is inside the range spanned by the epidemic scenarios. Otherwise, it is the RMSE between observed incidence and the incidence of the closest epidemic scenario, normalized to the range spanned by observed incidences in the respective country. The order and color of the bars and segments is identical, that is the bars serve as color legend for the segments. As orientation: the leftmost dark bar corresponds to the dark segment with the percentage information inside, they represents the number or percentage of countries with the smallest nRMSE.

To assess the prediction error of our workflow, we compute the normalized root mean square error (nRMSE) between prediction scenarios and observations. The nRMSE is zero, if the observation lies in between the simulation scenarios. Otherwise, the nRMSE is the RMSE between observation and the closest prediction scenario, normalized to the range that is spanned by the observations in the respective target country (for details, see Materials and Methods section). Fig. 4 captures the absolute and relative frequency of countries according to their nRMSE. Our predictions are in very good agreement (nRMSE=0) for Alpha in 81.4%, B.1.1.529 in 53.1%, BA.2 in 52.9%, BA.5 in 49% and for Delta in 12.7% of all considered countries. Note that even though Delta has the smallest amount of countries with incidences falling within scenarios prediction, more than 75% of the countries have a nRMSE≤2.5.

*Alternative Step 3 and 4: The pandemic delay as a simple integrative measure for variant classification*. Despite the simplicity of the projection approach, the numerical simulation on country level makes it difficult to summarize an emergent variant’s pandemic potential in simple terms. To close this gap, we introduce the pandemic delay $t_{y}$, that combines phylogenetic, connectivity and epidemic information in a single equation by assuming that the new variant has a fitness advantage Δf against the pre-existing strains and is competing for the infected population estimated via a simple logistic growth equation (see Materials and Methods section for a detailed derivation). The pandemic delay $t_{y}$ estimates the time between tMRCA and when the new variant reached a fraction *y* of all sequenced probes in the target country *m*:


(4) $$t_{y}(m)=-\frac{1}{\Delta f}\ln\;\left(\frac{1-y}{[1/x_{0}(m)-1]y}\right).$$

**Fig. 5.**
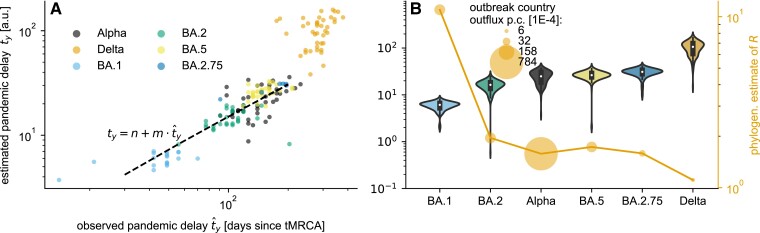
Pandemic delay. The pandemic delay $t_{y}$ until the lineage reaches a fraction y∈[0.13,0.16] is estimated for Alpha, Delta, BA.1 (B.1.1.529), BA.2, BA.2.75, and BA.5. A) The country-specific estimates of $t_{y}$ versus the observed pandemic delay ${\hat{t}}_{y}$ suggest a linear relation, highlighted by a Theil-Sen estimation (dashed line) with slope m≈1.55 and intercept n=−0.47 based on all but the Delta lineage data. B) The lineages sorted according to their median $t_{y}$ in combination with the phylogenetic estimate of the reproduction number *R* show that the additional connectivity information, the monthly outbreak country outflux per capita (equivalent to the export probability), explains the shorter predicted pandemic delay of the Alpha lineage despite its second-lowest median *R*.

The phylogenetic information is encoded in the fitness advantage Δf=lnR−ln1 with *R* as the phylogenetic estimate of the reproduction number, that is we assume that the population behaviorally and/or medically adapted to the pre-existing strains resulting in a $\hat{R}=1$. The initial fraction $x_{0}(m)$ encodes the connectivity between outbreak and target country and their epidemic state, that is it estimates how many cases are at tMRCA imported relative to the current case number. Fig. 5A shows a qualitative agreement between our estimated $t_{y}$ and the observed pandemic delay ${\hat{t}}_{y}$ (r≈0.85, p≪0.001) and suggests a linear relation considering all but the Delta lineage’s overestimated delay. Note that also within the lineages, the correlation between estimated and observed delay is in general high and significant ([r-, p-value]: Alpha [0.5,0.001], Delta [0.3,0.06], BA.1 [0.52,0.02], BA.2 [0.14,0.47], BA.2.75 [0.97,0.002], BA.5 [0.44,0.008]), which highlights the importance of the additional connectivity information. The rank correlation between median estimated $t_{y}$ and the phylogenetic estimate of *R* (Fig. 5B) is almost perfect, with the Alpha lineage as an exception that has a shorter pandemic delay (rank 2) than expected if solely *R* would be considered (rank 4) because of the particularly high outflux per capita of its outbreak country (Great Britain). Again, it illustrates that the combination of all information is necessary to gain a realistic estimate of an emergent lineage’s pandemic potential.

## Discussion and conclusions

We presented an integrated framework that combines phylogenetic analysis of genomic surveillance data with international human mobility data and large-scale epidemic modeling, in order to characterize in nearly real time the pandemic potential of an emerging variant. This concept is intended to provide quantitative indicators about the ability of a variant to escape population immunity acquired by previous infections and/or vaccination, and quickly spread at a global level through human activities.

Our framework naturally deals with missing and noisy information to infer, through a Bayesian approach, the most likely origin—in space (on the country level) and time—of an emerging variant and its growth rate. Spatial and temporal coordinates are used to feed an analytical technique to estimate the probability that a given number of infectious individuals, departing from the country where the variant first appeared, travel to other countries with no exposure to it. This crucial step is based on international travel data, providing information about human movements between countries. Note that our approach is more powerful than naive estimates based only on origin–destination pairs: in fact, we make full use of the knowledge we have about the underlying international travel network and its latent geometry (22, 45, 44), known to play a crucial role to amplify the spread of an emerging pathogen (19). The last stage of our framework is to use importation risk to quantify the number of imported infectious cases to each country and, accordingly, estimate the consequent unfolding of the epidemic due to the emerging variant. The epidemic model is intended to quantify undetected infections that occur well before the first genomic sequence is isolated from a case in a country. Finally, we validate our estimate of the pandemic delay, that allows a simple to interpret qualitative comparison between variants incorporating phylogenetic, epidemic, and connectivity information. The estimation is based on a logistic growth equation for the relative fraction of a new variant. These predictions will be less accurate if growth advantages in different countries are heterogeneous, for example, due to immune escape.

Only the early phase of spread of a new lineage is estimated and the proposed model can safely take advantages of assumptions like a homogeneous mixing and the lack of feedback loops in the epidemic dynamics.

We have validated our integrated framework on existing variants, including B.1.1.7 (Alpha), B.1.617.2 (Delta), B.1.1.529 (BA.1), BA.2, BA.2.75, and BA.5 (Omicron), finding excellent agreement with independent estimates of the relevant phylogenetic and epidemiological parameters. By accounting for different scenarios in the progress of the epidemic in each country, we provide quantifiable indicators to inform decision makers and support pro-active policy interventions to mitigate the potentially harmful effect of an emerging variant, as preventing a sudden overburden of the national health care system. For the variant of most concern at the time of writing this manuscript (early 2023), BA.5, we estimate that its most recent common ancestor existed in early January 2022 (January 10, 2022, 95% HPD: December 19, 2021–January 29, 2022), with a daily growth rate of 0.110 (95% HPD: 0.051–0.177).

Overall, our findings show that it is possible to aim at pandemic intelligence, even with partial and noisy data. We must caution that the estimates of the pandemic potential of an emerging SARS-CoV-2 variant are largely driven by the uncertainty in the spatio-temporal coordinates of its origin. The Delta lineage is our most unreliable estimate (Figs. 3–5), possibly due to the low-genomic surveillance at the tMRCA in the outbreak country India, even if Delta and Alpha have a comparable sequencing rate corrected for under-reporting (Supplementary Table S1), because the under-reporting is based on COVID-mortality that is known to be again underestimated in India by a factor of 6 to 7 (46). Another reason especially for the overestimation of Delta’s pandemic delay (Fig. 5) is its low phylogenetic growth rate estimate, which implies that during the long time till Delta dominates, additional mutations can happen that potentially speed up the process. Importantly, note that only the validation of our scenario predictions relies on large enough sequencing rates in the target country, but not its application. That means our framework is perfectly suitable for low- and middle-income countries with little to no genomic surveillance, as long as disease related mortality is monitored.

Our framework relies on the country of origin’s capacity to sequence a fraction of its positive tests for the phylogenetic analysis. Thus, it is crucial to support international efforts that enhance the diagnostic capabilities of countries (48, 47). However, in case the outbreak country can differentiate between SARS-CoV-2 variants by other means, for example high-throughput PCR assays (49, 50), the alternative characterization of the growth rate presented in Step 1 by epidemic modeling can be applied. Additionally, the country of origin can be reassured by the outbreak-origin-detection analysis, as described in Step 2.

Failures in international cooperation with a view to finding global solutions have undoubtedly shaped the COVID-19 pandemic. We have provided robust evidence that epidemic intelligence at country level could be not enough, alone, to contrast the pandemic of respiratory pathogens such as SARS-CoV-2, in the absence of well-coordinated genomic surveillance—especially in low- and middle-income countries currently lacking an adequate response capacity (51)—and global projections of variant’s pandemic potential. Our approach is inherently integrated and scalable, adding to ongoing modeling efforts and pan-viral analyses (52, 53, 55, 54, 23, 11) and responding to global calls for coordinated action (51, 57, 56). The data-driven approach provides a vital step in the path towards pandemic intelligence—where the interconnected and interdependent nature of human activities (22, 19, 58) is naturally accounted for at a global level—as well a means of enhancing global preparedness against future emerging variants.

## Acknowledgments

The authors acknowledge the GISAID initiative and all the authors from the originating laboratories where genetic sequence data were generated for sharing such data through GISAID (34), which has made this work possible. We further are thankful and acknowledge the CoVariants.org project (59) and *Our World in Data* (60). The authors thank the anonymous reviewers for their valuable suggestions.

## Supplementary Material

pgad192_Supplementary_Data

## Data Availability

The data and code to run the analysis are publicly available on Zenodo in https://doi.org/10.5281/zenodo.7998143. Regarding the worldwide transportation data, the repository contains (i) the monthly computed import probabilities between January 2020 and June 2022 and (ii) a subset of the world air-transportation network (WAN) with shuffled and randomized links to test the import risk code, since publicly sharing the WAN-data is not permitted by the distributing company OAG (https://www.oag.com/airline-schedules-data). For details on the WAN, please contact dirk.brockmann@hu-berlin.de.

## Previous presentation

These results were not previously presented.

## Preprints

A preprint of this article is published at 10.1101/2022.08.19.22278981.
