# Supplementary material for: Enhancing global preparedness during an ongoing pandemic from partial and noisy data
Source: PNAS Nexus. 2023 Jun 7;2(6):pgad192. doi: 10.1093/pnasnexus/pgad192 (PMC10282504; doi:10.1093/pnasnexus/pgad192)
Supplement: pgad192_Supplementary_Data [file pgad192_supplementary_data.pdf]

SI Appendix
Material and Methods and Supplementary Information
"Enhancing global preparedness during an ongoing pandemic from partial and noisy data"

Pascal Klamser[1,2,†,*], Valeria d'Andrea[3,†], Francesco Di Lauro[4], Adrian Zachariae[1,2], Sebastiano Bontorin[3,5], Antonello di Nardo[6], Matthew Hall[4], Benjamin F. Maier[1,2], Luca Ferretti[4], Dirk Brockmann[1,2], Manlio De Domenico [7,8,*]

[1]Robert Koch-Institute, Nordufer 20, 13353 Berlin, Germany
[2]Institute for Theoretical Biology, Humboldt-University of Berlin, Philippstr. 13, D-10115 Berlin, Germany
[3]Fondazione Bruno Kessler, Via Sommarive 18, 38123 Povo (TN), Italy
[4]Big Data Institute, University of Oxford, Li Ka Shing Centrefor Health Information and Discovery, Oxford, UK
[5]Department of Physics, University of Trento, Via Sommarive 14, 38123 Povo (TN), Italy
[6]The Pirbright Institute, Ash Road, Pirbright, Woking, Surrey, United Kingdom
[7]Department of Physics and Astronomy, G. Galilei,University of Padua, Via Francesco Marzolo 8, 35131, Padua, Italy
[8]Padua Center for Network Medicine, University of Padua, Via Francesco Marzolo 8, 35131, Padua, Italy
[†]Contributed equally to this work.
[*]Corresponding author. E-mail: manlio.dedomenico@unipd.it, klamser@physik.hu-berlin.de

Contents

I	Phylogenetic Reconstruction		S2
	I.1	Genomic dataset compilation	S2
	I.2	Phylogenetic estimates of epidemiological parameters	S2
	I.3	Estimates based on epidemic modeling	S2
II	Import Risk estimation		S5
	II.1	International travel dataset compilation	S5
	II.2	Quantifying the Import Risk	S6
	II.3	Relation to distance and arrival time	S7
	II.4	Alternative distance measures	S8
	II.5	Data for arrival time and outbreak region	S9
	II.6	Outbreak detection based on 1st count GISAID data	S9
III	Epidemic Scenarios		S12
	III.1	Renewal equation	S12
	III.2	A fully worked out example: the Alpha variant	S13
	III.3	Prediction error	S14
IV	Pandemic delay		S17
V	Information Distance		S19

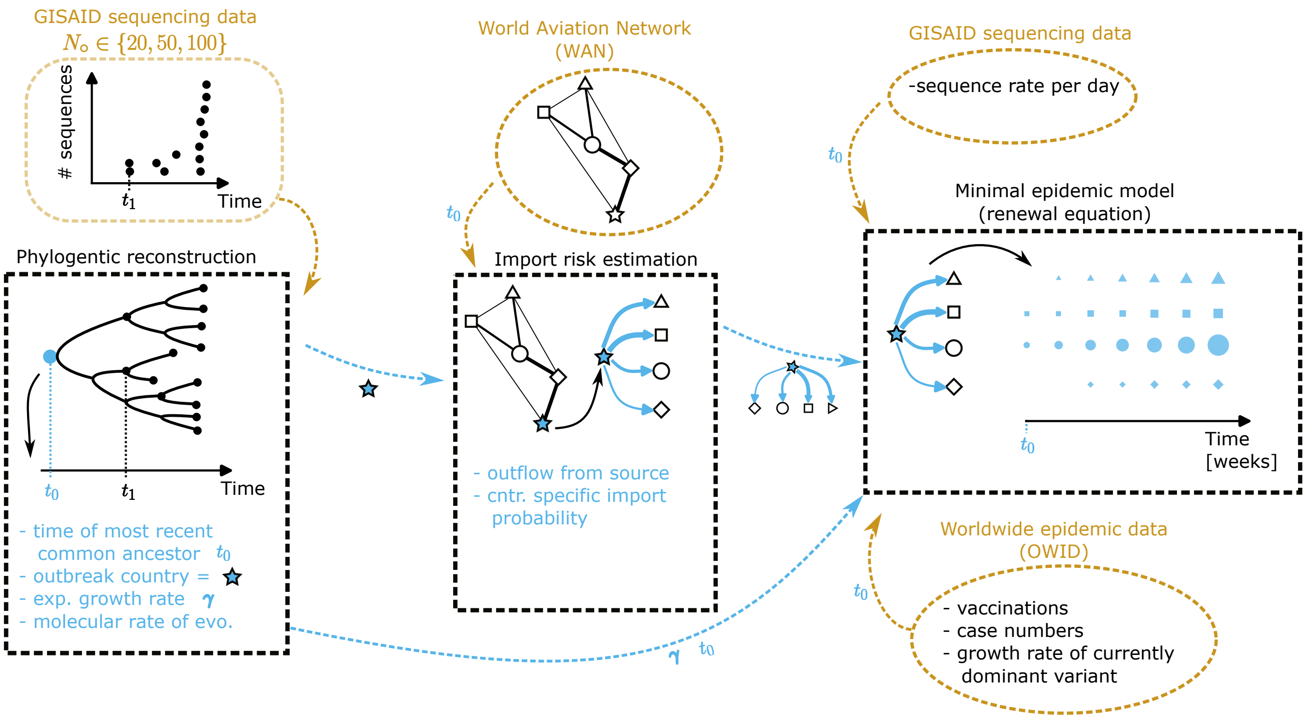

Fig. S1. Schematic mechanistic pipeline workflow. The three pieces of our pipeline (black dashed boxes) are illustrated and what input they get from external data sources (orange colored) or from the output of earlier parts of the pipeline (blue arrows). The t_0 close to the orange arrows means that data from the external sources is used at or prior to t_0, which is the estimated time of the most recent ancestor (the output of the first part of the pipeline, the phylogenetic reconstruction).

Materials and Methods

Phylogenetic Reconstruction

Genomic dataset compilation

We retrieved all SARS-CoV-2 sequences belonging to the Alpha B.1.1.7, Delta B.1.617.2, Omicron B.1.1.529 (BA.1), BA.2, BA.5, and BA.2.75 lineages from GISAID. Each genomic dataset was filtered by only retaining those sequences that were generated from cases reported during the initial wave and from the country of evolutionary origin, up to a total of 100 sequences per lineage. We then generated 3 alignments using MAFFT 7.505 [1], each comprised of 20%, 50% and 100% of the total number of sequences, which were subsequently cleaned by trimming the $5'$ and $3'$ untranslated regions and gap-only sites.

Phylogenetic estimates of epidemiological parameters

We performed a common Bayesian evolutionary reconstruction of timed phylogenetic history using BEAST 1.10.5 [2] that was source compiled from its GitHub repository (`https://github.com/beast-dev/beast-mcmc`). We modelled the nucleotide substitution process according to a $HKY85{+}\Gamma$ parameterisation, setting a strict molecular clock and an exponential growth model as coalescent prior. We used a $Lognormal(\mu = 9 \times 10^{-4}, \sigma^2 = 1 \times 10^{-5})$ prior for the molecular rate of evolution, a $Laplace(\mu = 0, b = 100)$ prior for the rate of exponential growth and a $Lognormal(\mu = 5.7, \sigma^2 = 2.3)$ prior for the exponentially growing viral population size. We further set an initial calibration for the time of the most recent common ancestor (tMRCA) at an age of $\sim$ 6 months before the most recent sample included in the alignment. All the remaining priors were left at their default values.

Bayesian inference through Markov chain Monte Carlo (MCMC) was performed for 2×10^8 generations, sampling every 20,000 generations and using the BEAGLE 3.1.2 library to increase computational performance [3]. MCMC convergence and mixing properties were inspected using Tracer 1.7.2 [4] to ensure that effective sample size (ESS) values associated with estimated parameters were all >200. After discarding 10% of sampled trees as burn-in, estimates of the growth rate, molecular clock and tMRCA were extracted along with their posterior distributions (Figure S2).

Estimates based on epidemic modeling

We obtain an independent estimate for t_0, the time of the first unreported case, and for other epidemic parameters, such as the effective reproduction number and the generation interval. By indicating with $I(t)$ the number of infected individuals at time t and with $D(t)$ the number of deaths, we consider the stage with the co-circulation of an existing variant v and the emerging one ω. Since we consider the final stage of the contagions due to v and the early stage of the contagions due to ω, we approximate the

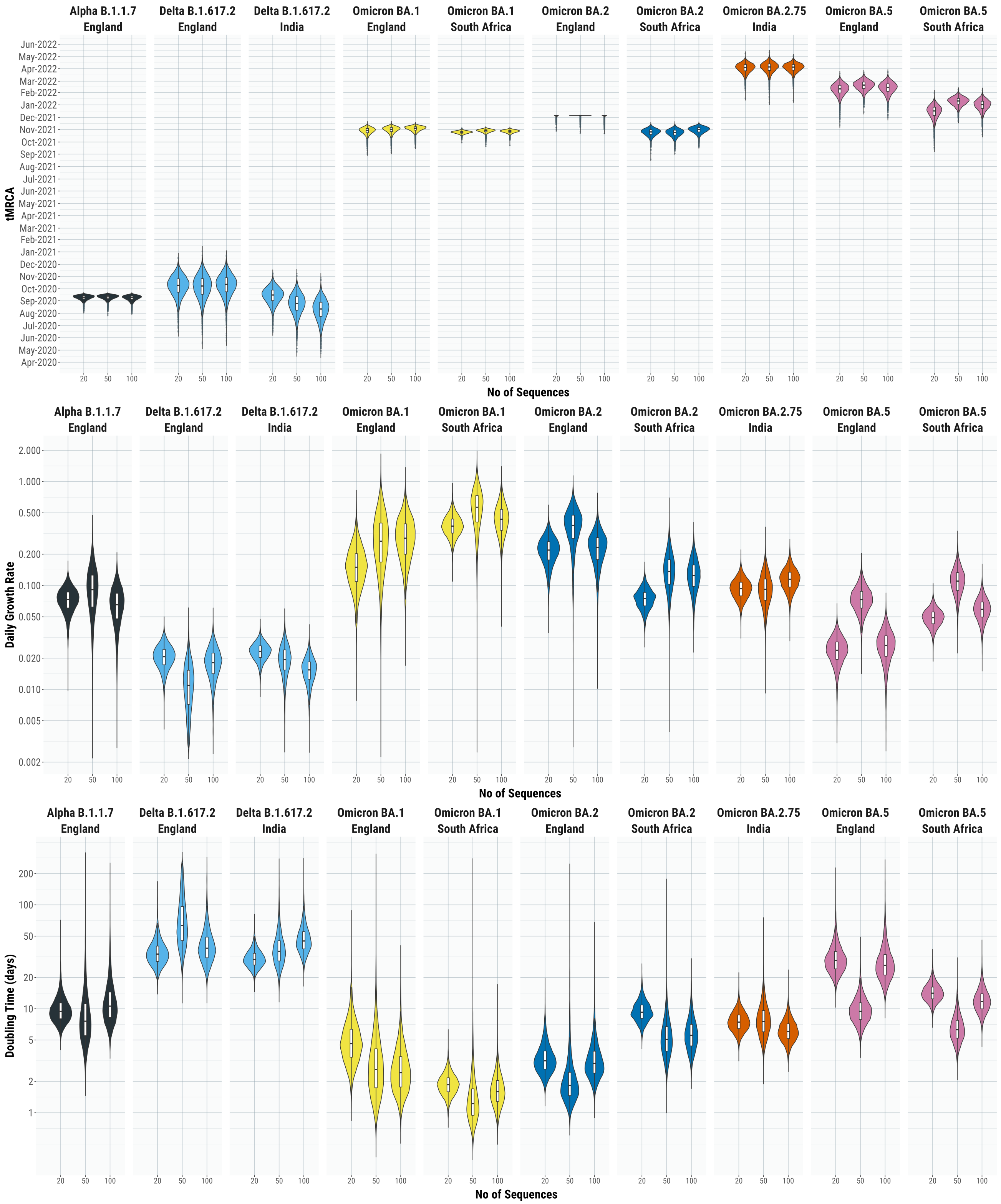

Fig. S2. Pan-variant phylogenetic analysis. Posterior distributions of the time of the most recent common ancestor (tMRCA), daily growth rate and doubling time estimated for each of the Alpha B.1.1.7, Delta B.1.617.2, Omicron B.1.1.529 (BA.1), BA.2, BA.5, and BA.2.75 SARS-Cov-2 lineages using alignments of 20, 50 and 100 sequences.

epidemic evolution by

$$\begin{aligned} I(t0+\Delta t) &= I_v(t0+\Delta t) + I_\omega(t0+\Delta t) \\ &= I_v(t_0)R_v(t_0)^{\Delta t/\mathrm{GI}_v} + I_w(t_0)R_w(t_0)^{\Delta t/\mathrm{GI}_\omega}, \end{aligned} \tag{S1}$$

where $I_x(t)$ is the number of infections due to variant x at time t, $R_x(t_0)$ is the effective reproduction number at time t_0 and GI_x is the generation interval. Similarly, the deaths due to the co-circulating variants are approximated by

$$D_v(t0 + \Delta t + \tau_v) = I_v(t0 + \Delta t) \times \mathrm{IFR}_v, \tag{S2}$$

$$D_\omega(t0 + \Delta t + \tau_\omega) = I_v(t0 + \Delta t) \times \mathrm{IFR}_w, \tag{S3}$$

$$D(t) = D_v(t) + D_\omega(t) \tag{S4}$$

where IFR_x denotes the infection fatality rate of variant x and τ_x is the lag between infection and death. To fit the unknown parameters, i.e. the ones related to variant ω, we use particle swarm optimization [5] to minimize the loss function

$$\phi(\theta) = \frac{1}{2}\frac{\sqrt{\mathrm{Var}[\log(1 + I(t)) - \log(1 + I_{obs}(t))]}}{\sqrt{\mathrm{Var}[\log(1 + I_{obs}(t))]}} + \frac{1}{2}\frac{\sqrt{\mathrm{Var}[D(t) - D_{obs}(t)]}}{\sqrt{\mathrm{Var}[D_{obs}(t)]}}, \tag{S5}$$

where $I_{obs}(t)$ and $D_{obs}(t)$ are the number of infected individuals and deaths from empirical data [6], Var indicates the variance in time and $\theta = \{t_0; R_\omega(t_0); \mathrm{GI}_\omega; \mathrm{IFR}_\omega; \tau_\omega\}$ is the vector of the epidemiological parameters characterizing the emerging variant, for which we obtain a joint probability distribution.

Import Risk estimation

International travel dataset compilation

We retrieve the monthly seat capacities between airports from the OAG (Official Airline Guide). Note, that it does not represent the actual passengers that flew from airport A to B in one month, but the maximal capacity, i.e. how many could have travelled if all seats were occupied. It is therefore an upper limit for the passenger flux and we refer to it as the flow matrix $\mathbf{F}$, where F_{ij} describes the maximal passenger flow to i from j. We estimate the travelling population in the catchment area of an airport by $N_i = F_i$, with $F_i = \sum_j F_{ji}$, i.e. we assume that the population is proportional to the outflux of the airport. For each variant, we use the world air-transportation network (WAN) at the month of the outbreak day of the respective variant.

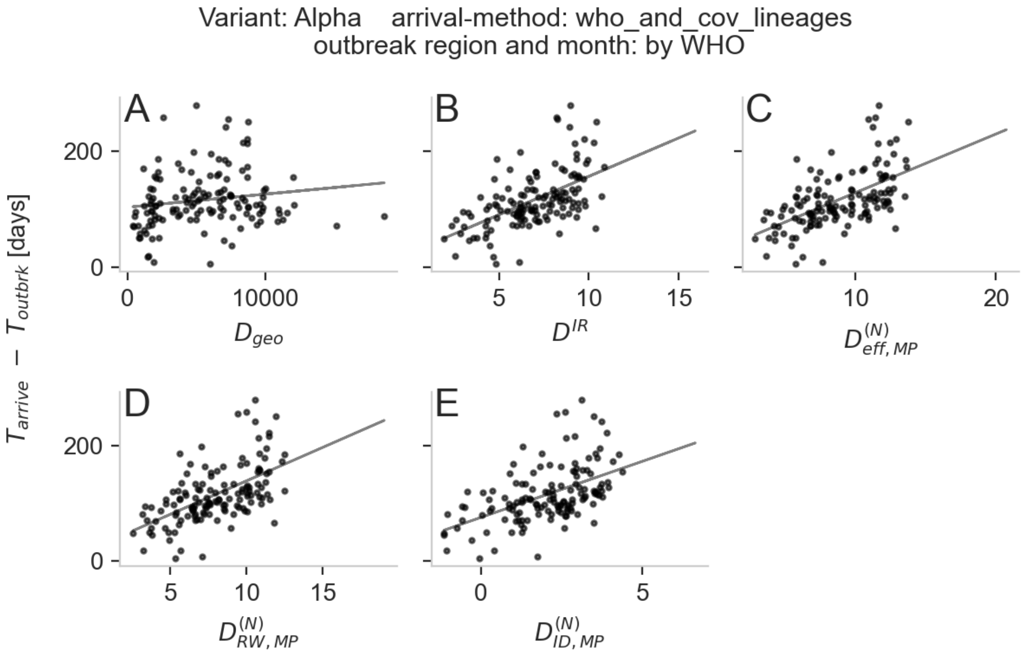

Fig. S3. Distance measures vs. arrivals for Alpha variant. The distance measures are the geographic distance D_{geo} (**A**), the import risk distance D^{IR} (**B**), the effective distance $D^{(N)}_{eff,MP}$ (**C**), the random walk distance $D^{(N)}_{RW,MP}$ (**D**) and the information diffusion distance $D^{(N)}_{ID,MP}$ (**E**) whereby the latter three (**C, D, E**) are generalized to weighted multiple paths.

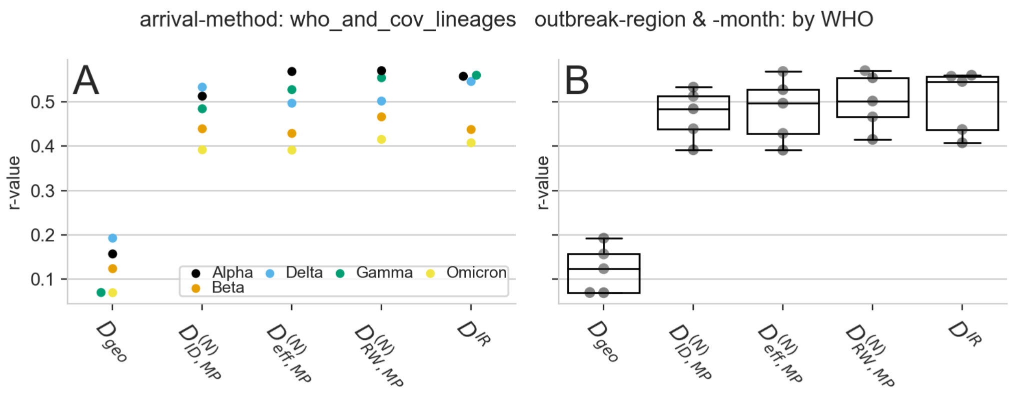

Fig. S4. Correlation comparison between different distance measures. The distance measures are the geographic distance D_{geo}, the import risk distance D^{IR}, the effective distance $D^{(N)}_{eff,MP}$, the random walk distance $D^{(N)}_{RW,MP}$ and the information diffusion distance $D^{(N)}_{ID,MP}$ whereby the latter three are generalized to weighted multiple paths.

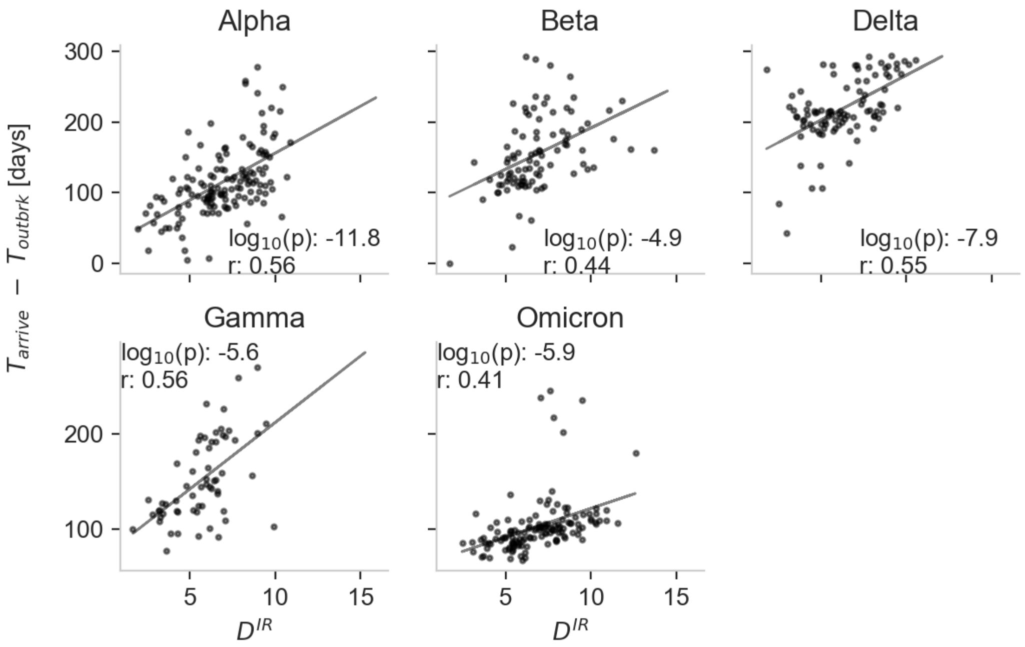

Fig. S5. Correlation of arrival times of variants with the import risk distance D^{IR}. For the import risk distance $D^{IR}(m|n_0) = -\log(p_\infty(m|n_0))$ the WAN of the WHO outbreak month is used and the WHO outbreak location as source country. The arrival times are taken from the "cov-lineages.org" [7, 8] project.

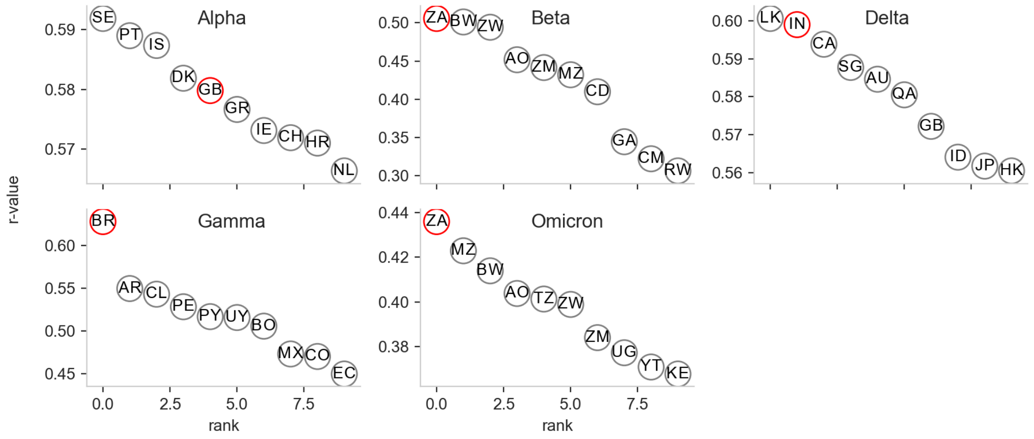

Fig. S6. Arrival prediction (r-value) for the 10 best outbreak candidate. The r-value between the import risk distance $D^{IR}(m|n_0) = -\log(p_\infty(m|n_0))$ and the arrival time for the 10 best ranked outbreak countries (n_0). The 2 Letters in the circles are the countries ISO alpha-2 codes. The red circle marks the country declared as outbreak country by the WHO.

Quantifying the Import Risk

The import risk method is introduced in a separate study [9] where it is compared to another data-driven estimate. Here we present a short outline of the method. To know how many passengers leave at node j given they started at node i, we introduce the shortest path exit probability $q(j|i)$ (SPEx). It is based on the shortest path tree of the effective distance [10], and combines the exit probability with all possible paths that end in j. The resulting import risk is therefore an extension of the SPEx.

In order to compute the **SPEx** we first define, with the flow matrix (maximal passenger flux) $\mathbf{F}$ and the travelling population of the catchment area N_i, the transition matrix $\mathbf{P}$, where the element $P_{ij} = F_{ij}/\sum_i F_{ij} = F_{ij}/F_j$ is the probability to transition to i from j. Now, the effective distance graph [10] is $D_{ij} = d_0 - log(P_{ij})$, with d_0 as the distance offset which we set to $d_0 = 1$ (the larger d_0 the more D_{ij} increases with increasing hop-distance). Let $\mathbf{T}(n_0)$ be the shortest path tree on $\mathbf{D}$ for the point of origin n_0. With respect to node n the downstream nodes $\Omega(n|n_0)$ are those nodes that can be reached from the source n_0 through node n on $\mathbf{T}(n_0)$.

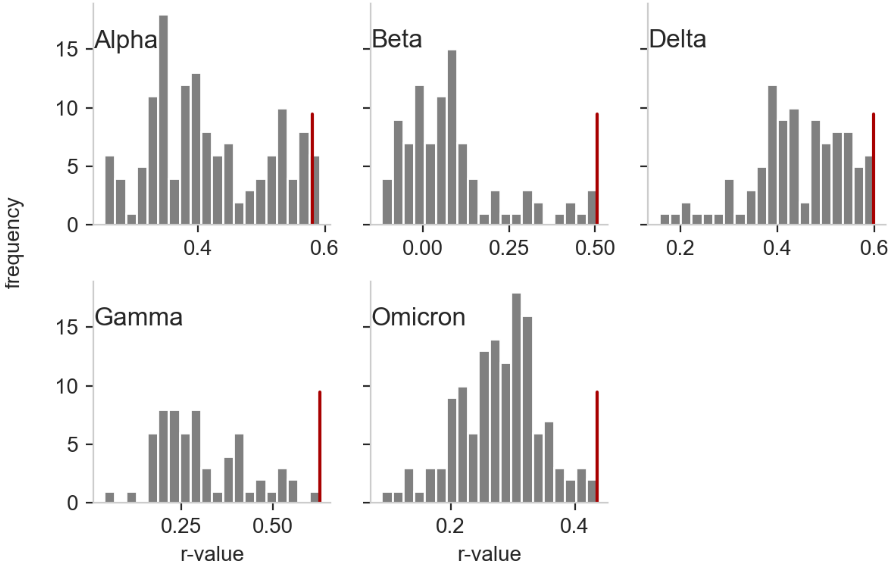

Fig. S7. Arrival prediction performance (r-value) for the outbreak country candidates. The frequency of the r-value between the import risk distance $D^{IR}(m|n_0) = -\log(p_\infty(m|n_0))$ and the arrival time for all possible outbreak countries. The red vertical line marks the r-value using the country declared as outbreak country by the WHO.

Now we compute the SPEx $q(i|n_0)$ by assuming that all passenger that start at n_0, travel along the shortest path tree $\mathbf{T}(n_0)$ and distribute to other airports according to their respective populations N_n. We assume that the exit probability at i is proportional to the ratio of the population at i (i.e. N_i) to the population of all of i's downstream nodes $\sum_{n\in\Omega(i|n_0)} N_n$ plus N_i:

$$q(i|n_0) = \frac{N_i}{N_i + \sum_{n\in\Omega(i|n_0)} N_n} \ . \tag{S6}$$

Now, we use the SPEx on a random walk that starts at n_0 and the walker exits at node i with probability $q(i|n_0)$ or continues its walk with probability $1 - q(i|n_0)$. Thus, the probability to be at node m if the walker was before at node $m-1$ is

$$S(m, m-1|n_0) = P_{m,m-1}(1 - q(m-1|n_0)) \ . \tag{S7}$$

Consequently, the probability to take a path Γ starting at n_0 and exiting at m is

$$p(\Gamma) = q(m|n_0) \prod_{(i,j)\in\Gamma} S(i,j|n_0) \ . \tag{S8}$$

The probability to exit at node m from all possible paths (of all possible lenghts) is

$$p_\infty(m|n_0) = q(m|n_0) \left[\sum_{k=1}^{\infty} \mathbf{S}^k(n_0)\right]_{m,n_0} \tag{S9}$$

$$= q(m|n_0) \left[(\mathbf{1} - \mathbf{S}(n_0))^{-1} - \mathbf{1}\right]_{m,n_0} \ . \tag{S10}$$

Note that $\mathbf{S}^k(n_0)_{m,n_0}$ is the probability sum of all paths that started in n_0 and end after k steps in m. We aggregate all airports of the same country by computing the weighted mean with weights

$$w_n = \frac{N_n}{\sum_{m\in C(i)} N_m} \tag{S11}$$

with $C(n)$ as the set of airports that belong the same country as node n does.

Relation to distance and arrival time

In order to assess the quality of the import risk, we compare it with the arrival time of past variants. Clearly, the higher the import risk to a country, the earlier it is to arrive and the direct relation between the probability of travel to a city m from a city n_0 and

the mean first arrival time t_1 is

$$t_1(m|n_0) = d_0 - c\log(P(m|n_0)) \tag{S12}$$

which is the effective distance [10, 11]. Thus, we define the import risk distance as

$$D^{IR}(m|n_0) = -\log(p_\infty(m|n_0)) \tag{S13}$$

which is proportional to the mean first arrival time.

Alternative distance measures

There are alternative measures to estimate the arrival time [10, 12, 13], and we want to compare our import risk distance to these established measures. However, please note that the alternative measures have a clear qualitative relation to the arrival time, but it is not possible to directly infer the number of passengers that travel between airports from them (what the import risk is especially designed for). The already introduced alternative measure is the effective distance [10] that uses the flow between airports to estimate the probability to travel from airport n to m

$$d_{eff}(m,n) = d_0 - \log(P_{m,n})\ . \tag{S14}$$

Now, the distance along a specific path Γ that connects m and n_0 is the sum of the path elements distances

$$d_{eff}(\Gamma) = \sum_{(m,n)\in\Gamma} d_{eff}(m,n)\ . \tag{S15}$$

Finally the effective distance from airport n_0 to m, also not directly connected airports, is the minimal effective distance of all possible paths $\Omega(m,n_0)$ they are connected through

$$D_{eff}(m|n_0) = \min_{\Gamma\in\Omega(m,n_0)}(d_{eff}(\Gamma))\ . \tag{S16}$$

An extension to the effective distance is the random-walk effective distance [13] that considers all possible paths connecting two airports $\Omega(m,n_0)$ instead of only taking the dominant path with the shortest distance:

$$D_{RW}(m|n_0) = -\ln\left(\sum_{\Gamma\in\Omega(m,n_0)} e^{-d_{eff}(\Gamma)}\right)\ . \tag{S17}$$

Note that the sum of path distances via their exponential is due to the linkage to the arrival time as explained in [13].

We also add a comparison with a metric derived from Diffusion Distance [12] which exploits the definition of a random walk Laplacian on top of the WAN. We further explain this Information Distance D^{ID} in the dedicated section V.

Country-Level aggregation.

The country-level aggregation of the import risk distance D^{IR} is done by first aggregating the import risk on country-level (as described in Sect. II.2) and then applying Eq. S13.

To aggregate the other distances (D_{eff}, D_{RW}) we could either take (along the line of D_{eff}) the minimal distance between two countries (of all relevant airport pairs), or use a weighted multipath approach as used in the derivation of D_{RW}. We will highlight the latter in the following; however, we also computed the minimal measure and found that it is outperformed by the multipath distance (not shown, but it is the basic finding in [13]).

As shown in [11], the effective distance of two paths combined is

$$e^{-D_{eff}(\{\Gamma_a,\Gamma_b\})} = e^{-d_{eff}(\Gamma_a)} + e^{-d_{eff}(\Gamma_b)}\ . \tag{S18}$$

Thus, the multipath (MP) effective distance that considers all shortest paths from country S to M is:

$$D_{eff,MP}(M|S) = -\ln\left(\sum_{m\in\boldsymbol{M},s\in\boldsymbol{S}} e^{-D_{eff}(m|s)}\right) \tag{S19}$$

with $\boldsymbol{M}$ as the set of all target airports in country M and $\boldsymbol{S}$ all source airports of country S.

Since the distance of source airports with a larger population are more important, we additionally weight the source airport with $w_i = F_i/\sum_{s\in\boldsymbol{s}} F_s$, which represents the probability of an infected to start in location n. Now, we compute the population weighted multipath effective distance by

$$D^{(N)}_{eff,MP}(M|S) = -\ln\left(\sum_{m\in\boldsymbol{M},s\in\boldsymbol{S}} w_s e^{-D_{eff}(m|s)}\right)\ . \tag{S20}$$

Note that the weighting for the effective distance can be reformulated to

$$D^{(N)}_{eff,MP}(M|S) = -\ln\left(\sum_{m\in\boldsymbol{M},s\in\boldsymbol{S}} w_s \prod_{k,l\in\Gamma_{m,s}} e^{-d_0}P_{k,l}\right) \tag{S21}$$

which corresponds to multiplying the probability to start at the source airport s to the first step of each path. Analogously the

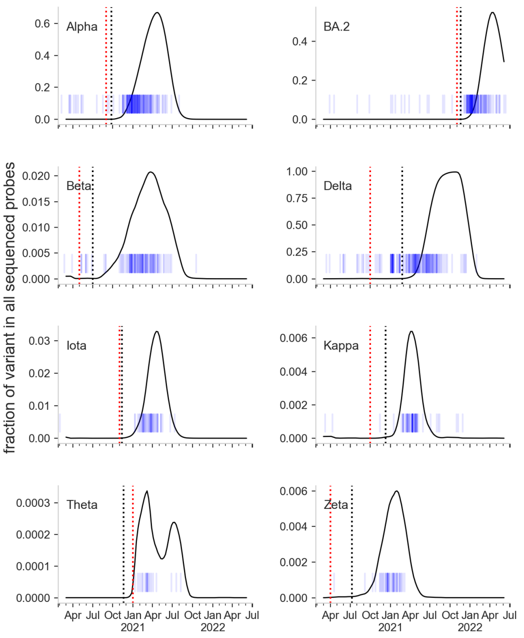

Fig. S8. Outbreak defined by fraction of all sequenced probes. The outbreak date (black dashed vertical line) of a variant can be defined by the first time the fraction of a variant X of all sequenced probes reaches 2.5% of its current worldwide peak. To exclude maldetections of 1st. arrival times in countries, we exclude all arrival times (blue short vertical lines) that are before the outbreak date and set the arrival time as the first detection in the respective country after the outbreak date. The official outbreak date by WHO is marked by a red dashed vertical line.

Data for arrival time and outbreak region

We compare the import risk to measured arrival times of different variants. Therefore, we need to define the outbreak-country and -month and the arrival times. We defined these variables in different ways.

(I) external sources Here we rely on peer reviewed [8] or official [14] sources. The outbreak country and the outbreak month are taken from the website of the World Health Organization (WHO) "Tracking SARS-CoV-2 variants"[14] and the arrival times of the variants Alpha, Beta, Delta, Gamma and Omicron were externally computed with "grinch"[8] and taken from their project website[7]. If arrival times are before the official outbreak they are removed from the analysis (for Delta=1, Gamma=1 and Omicron=19 countries are removed).

(II) GISAID data To also use the other variants to validate our import risk method we design a simple arrival time algorithm. First, we need to define the outbreak day. Instead of relying on an official definition from the WHO, we use GISAID data. The outbreak time $T_{X,out}$ of variant X is defined by

$$T_{X,out} = T\left(F_X(t) \geq g \cdot \max(F_X)\right) - 30\text{days} \tag{S22}$$

with $F_X(t)$ being the fraction of variant X to all sequenced probes at time t and $T(F_X(t) \geq g \cdot \max(F_X))$ the time when $F_X(t)$ crosses the first time the threshold $gcdot\max(F_X)$ where $g \in]0, 1[$ and we set $g = 0.025$. In other words, the outbreak is defined by 30 days before the variant reached 2.5% of its worldwide peak. We estimate the arrival time of variant X in an country by the most simple way: the first time the variant is detected (according to GISAID data). In Fig. S8 the estimated outbreak time, official WHO and arrival times of each country are shown. Since for some variants (Alpha, Delta, BA.2) many arrival times fall clearly before our estimated and even the official outbreak date, we recomputed for these countries the arrival time to the first GISAID-detection after the outbreak date. We argue that either (i) the sequencing of the variant in these countries was error-prone (1. count is very sensitive to any wrong detection) or (ii) the spreading was slow and the variant did not dominate the local epidemic until it reached a susceptible country (low NPIs) from where it did spread more easily (probably the case for Delta).

Outbreak detection based on 1st count GISAID data

If we repeat the outbreak detection method using all variants and the arrival times estimated via GISAID data (arrival by first detection, Fig. S8), we see that the outbreak detection via the best correlation between import risk distance D^{IR} and arrival times $T_{arrival}$ in general confirms the outbreak regions declared by the WHO (see Figs. S10, S9). There is a discrepancy for Delta. While using WHO and "cov-lineages.org" data, the official outbreak country India (IN) was second best, it is only on rank 12 if our GISAID estimates are used. A possible explanation is, that our outbreak date estimation is 5 months after the WHO date. In order to not lose the countries with arrivals before the outbreak date, we recompute the arrivals by the first count after the estimated outbreak date. One can argue that Delta did locally spread much stronger in South Africa (ZA, the top ranked country), and therefore is ZA for the worldwide distribution of larger importance than India. An alternative explanation is that the passenger flow in the WAN was too low and when it increased, ZA had a more active Delta epidemic.

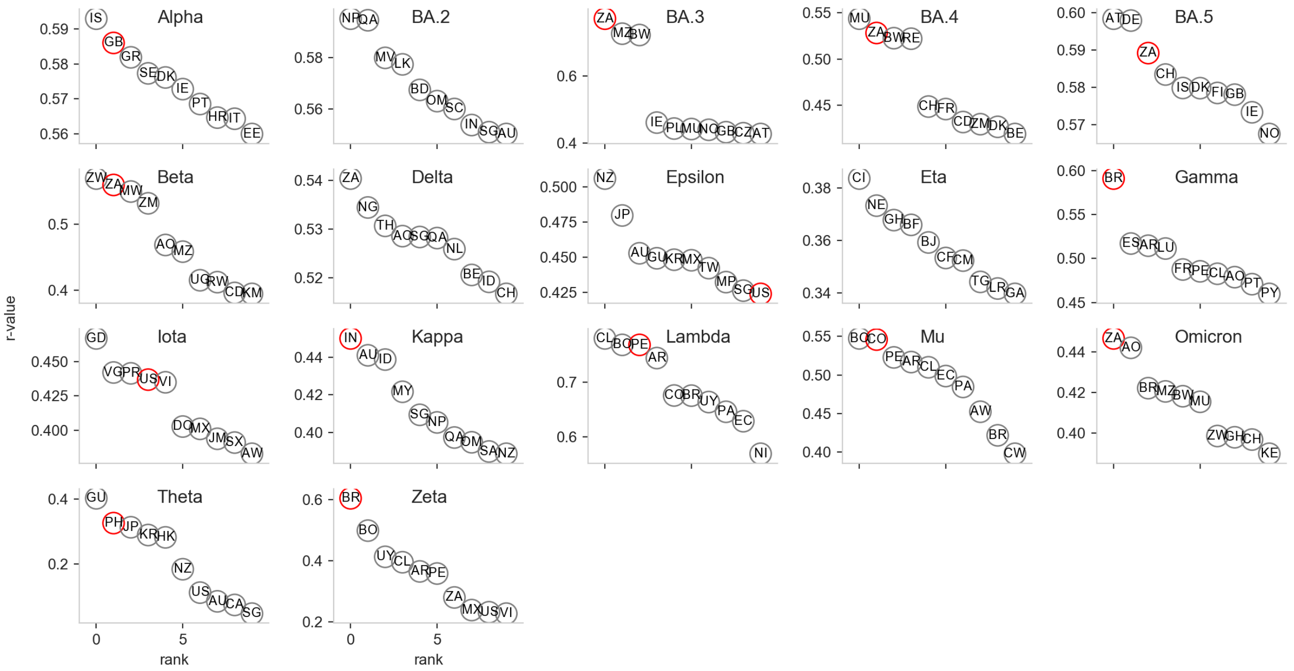

Fig. S9. Arrival prediction (r-value) for the 10 best outbreak candidate. The r-value between the import risk distance $d_\infty(m|n_0) = -\log(p_\infty(m|n_0))$ and the arrival time for the 10 best ranked outbreak countries (n_0). The 2 Letters in the circles are the countries ISO alpha-2 codes. The red circle marks the country estimated as outbreak country based on GISAID arrival times. In contrast to Fig. S6: the arrival times and outbreak dates are estimated via GISAID data (arrival by first count, outbreak date by reaching the first time 2.5% of worldwide peak of the respective variant).

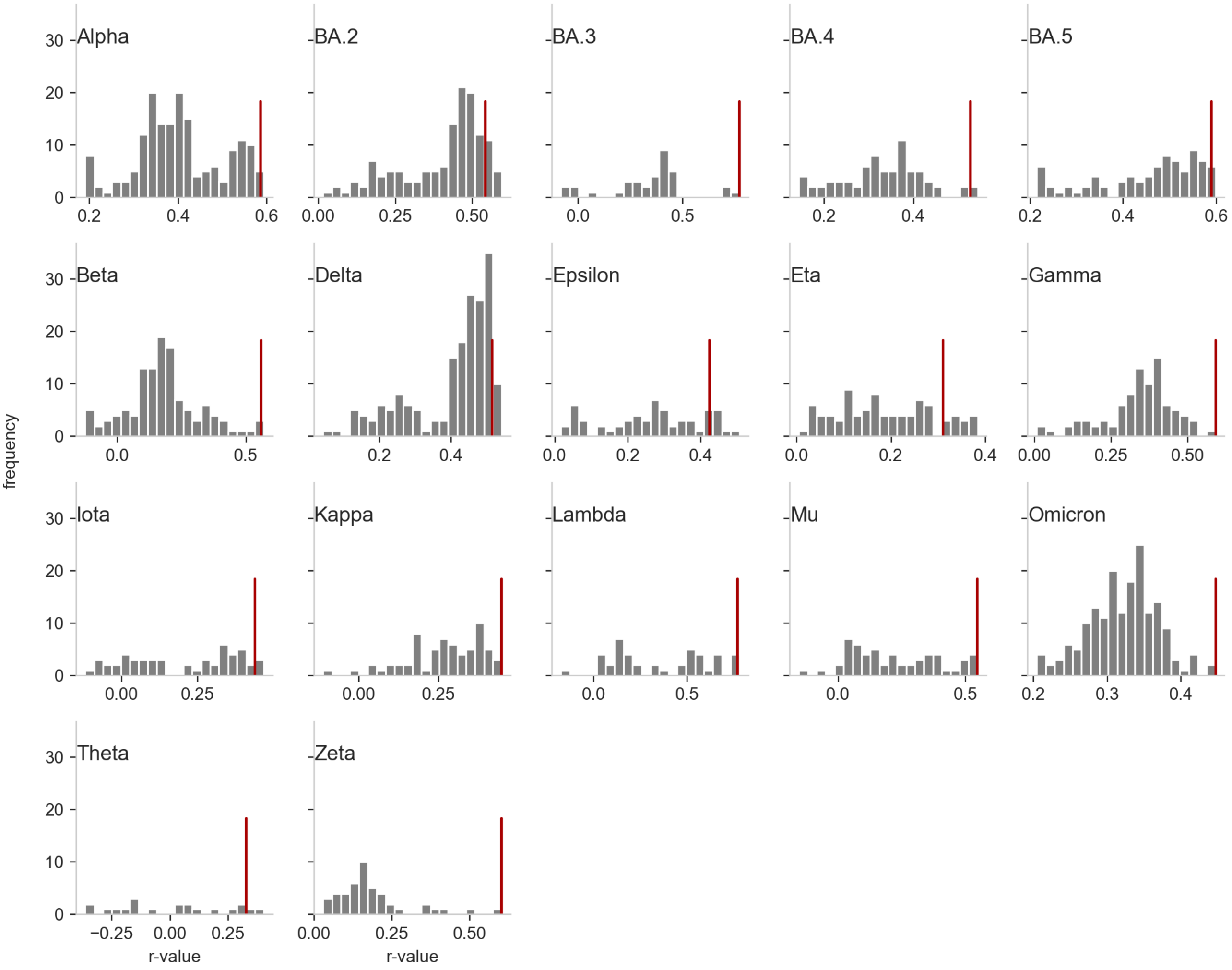

Fig. S10. Arrival prediction performance (r-value) for the outbreak country candidates. The frequency of the r-value between the import risk distance $D^{IR}(m|n_0) = -\log(p_\infty(m|n_0))$ and the arrival time for all possible outbreak countries. The red vertical line marks the r-value using the country estimated as outbreak country based on GISAID arrival times. In contrast to Fig. S7: the arrival times and outbreak dates are estimated via GISAID data (arrival by first count, outbreak date by reaching the first time 2.5% of worldwide peak of the respective variant).

Epidemic Scenarios

We consider two distinct models to project the number of daily new infected people, namely, a renewal equation based model and a multi-strain SIR-like model. The first one is actually part of the pipeline, while the second one is used as validation.

Renewal equation

The renewal equation approach is a well-known technique, widely used in epidemiology [15, 16, 17]. The reason why renewal equations are such strong candidates for early projection of new cases, is the fact that informing them requires only the reproduction number of the new variant of concern, its generation interval distribution, and the number of people infected by the new variant who travel into the target country from the source country. This allows easily to explore scenarios with different values of epidemiological quantities of interest, such as the effective reproduction number of a new variant as it spreads from the source country to others through travelers.

For now, we assume that the susceptible population is much larger than the number of active cases, and that the mixing between the infected and the susceptible is homogeneous. This allows to exclude feedback loops in the dynamics, e.g. the fact that immunity to the new variant builds up through infection, which would modify the dynamics itself. Such strong assumptions are acceptable as long as we restrict our projections to the very first few weeks from the introduction of the new variant in the target country.

The model assumes that the number of newly infected people at day t, $I(t)$, is given by two distinct processes: a) the arrival of infected individuals from the source country ($I_{out}(t)$) and b) the daily new infections ($I_{in}(t)$) happening in the target country due to the endogenous spreading. The former is estimated from section II, while the latter can be estimated through the renewal equation

$$I_{in}(t) = \sum_{s=t_0}^{t} \Gamma_s \mathcal{R}_s I(s), \tag{S23}$$

where t_0 is the day the first infected cases arrived in the target country, $\mathcal{R}_s$ is the daily reproductive number on day s, and Γ_s is the generation time distribution, i.e. the fraction of transmissions that would occur on day s after infection. Finally $I(t) = I_{out}(t) + I_{in}(t)$. This is the simplest renewal process, which does not include the fact that the target population might have an inhomogeneous immunological landscape, due to previous infections or vaccination. To model this phenomenon, we reinterpret the term on the right side of equation (S23) as the number of inoculations spreading from currently infecting people, which will turn into infections depending on the susceptibility of the recipients. If we assume that previous infections (with other variants) protect against reinfection with an efficacy of n_e, and, analogously, vaccination has an effectiveness of ν_e, then we can explicitly account for removals by modifying equation (S23) into

$$I_{in}(t) = \sum_{s=t_0}^{t} \Gamma_s \mathcal{R}_s I(s) \left(1 - n_e \frac{R^{(\text{old})}(t)}{N}\right) \left(1 - \nu_e \frac{V(t)}{N}\right), \tag{S24}$$

where $R^{\text{old}}(t)$ is the number of recovered people from previous variants that still have some protection against infections, and $V(t)$ is the total number of vaccinated people. This assumes that the number of recovered or vaccinated people is uniformly distributed across the population, and that the events 'being vaccinated' and 'having been infected' are independent. This also assumes no gradual waning of protection against infection. However, we can consider as recovered or vaccinated only people who were infected or vaccinated recently, rather than from the beginning of the pandemic. For instance, considering only people who got either infected or their second dose up to six months prior to t is equivalent to assuming that there is an abrupt waning of efficacy against protection six months after getting infected or vaccinated.

Although these hypotheses might seem unrealistic, the lack of readily available data about waning and immunological landscapes of various countries, and the fact that this should be used only for short-term scenario explorations, allow us to avoid introducing further complexity into the model.

The cumulative number of cases and amount of fully vaccinated individuals at each day are the ones reported in the public repository at [1]. We select the values for vaccine efficacy and protection from previous infection from available works. In particular we set the vaccine efficacy ν_e to 0 for Alpha, 0.5 for Delta, BA1 and BA2 and to 0.12 for BA.5 ([18, 19, 20, 21]). The selected protection against reinfection n_e is 1 for Delta, 0.56 for BA.1 and BA.2 Omicron lineages and 0.13 for BA.5 ([22, 23, 21]).

The second model is a multi-strain SIR inspired by [24]. This is a two-strain model in which people who recover after being infected with the former variant are not completely immune to infection from the latter variant. The equations governing this system are

$$\begin{cases} \frac{dS}{dt} &= -(\lambda_0(t) + \lambda_1(t))S(t) \\ \frac{dI^{(0)}}{dt} &= \lambda_0 S(t) - \gamma I^{(0)}(t) \\ \frac{dI^{(1)}}{dt} &= \lambda_1 S(t) + (1 - n_e\alpha)\lambda_1 R^{(1)}(t) - \gamma I^{(1)}(t) \\ \frac{dR^{(0)}}{dt} &= \gamma I^{(0)}(t) - (1 - n_e\alpha)\lambda_1 R^{(0)}(t) \\ \frac{dR^{(1)}}{dt} &= \gamma I^{(1)}(t) \end{cases} \tag{S25}$$

where $\lambda_i(t) = \beta_i \frac{I^{(i)}(t)}{N}$, β_i being the transmission rate of the variant i, and γ being the recovery rate. The initial condition $S(t_0), I_0(t_0), I_1(t_0), R_0(t_0), R_1(t) = \left\{S_0, I_0^{(0)}, I_{out}(t_0) + I_0^{(1)}, R_0^{(0)}, R_0^{(1)}\right\}$. Note that, since $I^{out}(t)$ represents the arrivals from the

[1] https://ourworldindata.org/

source country at the beginning of each day, the system is not closed. This is not a problem because we are considering countries, so $\frac{I^{out}(t)}{N} \ll 1$. Since the dynamics does not include, per se, the fact that the initial condition changes every day due to arrivals, we can solve this system on a daily basis, updating the initial condition and restarting the system accordingly. The advantage of this system is that it includes feedback phenomena, which is good when validating the model, as it may need to run for more than a few weeks. The drawbacks are that informing the model requires good point estimates of the various compartments, and the interpretation of the transmissibility coefficient related to the measured $\mathcal{R}_t$, which may not be straight-forward. For such reasons, this model is used to validate the renewal equation approach, in particular for countries where no new cases were observed after a few weeks from their emergence (not shown). Projections errors valuated with the SIR model relative to Alpha lineage are shown in

A fully worked out example: the Alpha variant

We apply our pipeline to a real case, the Alpha variant of concern (VOC), that was identified in the UK on 20 September 2020 [8]. We assume that the UK is the source country and we demonstrate how the pipeline works. In the following, we consider as the generation time interval distribution the one inferred from the literature [25].

Starting from the phylogenetic part of our pipeline, we take the time of emergence estimated when $n = 20$ sequences were collected, to simulate a realistic scenario where only little information is available. This gives a central estimate for the time of emergence of the Alpha variant around the 9th of November 2020. The daily growth rate estimated is $r = 0.097$ (95% HPD: 0.008–0.202). To translate this into R_t in the source country, we assume that all the growth rate advantage of Alpha relative to the previous circulating variants is given only by transmission advantage (limited capacity of reinfections with Alpha). Further, typical generation time distributions are Gammas, as in [25]. This allows us to estimate the R_t using formula 2.2 in [26]:

$$R = \frac{(r+b)^a}{b^a}, \tag{S26}$$

where b and a are the shape and rate of the Gamma distribution generation time. In our case, $a = 5.9, b = 1.13$, therefore $R_{t(\alpha)} = 1.62(1.04, 2.63)$.

For any target country, the projection of the number of cases infected with Alpha in the next weeks is performed in two steps: first, we estimate the number of infected travelers (referred to as seeds) who arrive in the target country from the source country, then we use the renewal equation (S24) on each possible scenario, to account for endogenous transmission of the secondary cases in the target country. The first step consists in using the import risk estimates described in section II to compute the number of daily travelers from source country to other target countries. We use import risk probability from source to target times the average daily outflow of passengers from source country using WAN data. We then determined the number of travelers infected with Alpha. This is done by considering the proportion of sequenced cases that are Alpha times the $7-day$ moving average of daily incidence of new cases, assuming that sequences are taken randomly from the infected population. This estimate does not include undercounting in the source country, which we can estimate as follows.

For a given country, we use the daily new estimated COVID-19 infections from the IHME model, which is a hybrid with two main components: a statistical "death model" component produces death estimates that are used to fit an SEIR model component [2]. For a complete overview of this model and a comparison with other estimates, we refer to OWID[3]. The data we used for our estimation are publicly available[4]. In a given temporal window, we integrate over time the confirmed number of cases (7d moving average) and the estimated true number of cases, as well as the estimates for its lower and upper bounds defining the 95% uncertainty interval. The mean undercounting factor is estimated by the ratio between the integrated estimate of the true cases and the confirmed ones in the temporal window, and similarly we estimate the corresponding uncertainty interval. We show in Fig. SS11 the undercounting factor obtained for all countries for which the data is available, whereas Fig. SS12 shows the evolution of this factor along periods of 6 months for some representative countries.

To allow for variability in undercounting, we consider two extreme scenarios: the best one, where undercounting is assumed to be 2.27, and the worst one, where undercounting is assumed to be 2.97. The number of infected travelers from the source country to the target country is then computed by multiplying the number of travelers into the target country by the proportion of infected people in the source country. This is often not a natural number. This is not a problem, as the renewal equation does not need to use integer number of infected people, and we interpret this as the results of the various averaging performed through all the steps. The model produces the total number of infected people in the target country given the seeds and the $\mathcal{R}_t$ by day of infection. To validate the model, we need to estimate how many people infected with the VOC were present in the target country during the considered period. We do so in the same way we estimate prevalence in the source country: by multiplying the proportion of sequenced cases that turned out to be Alpha times the daily incidence in the target country, scaled by the estimated undercounting factor.

The total number of different scenarios computed is, in this case $2 \times 2 \times 3$: undercounting in both the source and the target countries, and the different reproduction number of the VOC. Results are shown in Figure 3C and in Figure S13A.

[2] `https://covid19.healthdata.org/`

[3] `https://ourworldindata.org/covid-models`.

[4] `https://ourworldindata.org/grapher/daily-new-estimated-covid-19-infections-ihme-model`.

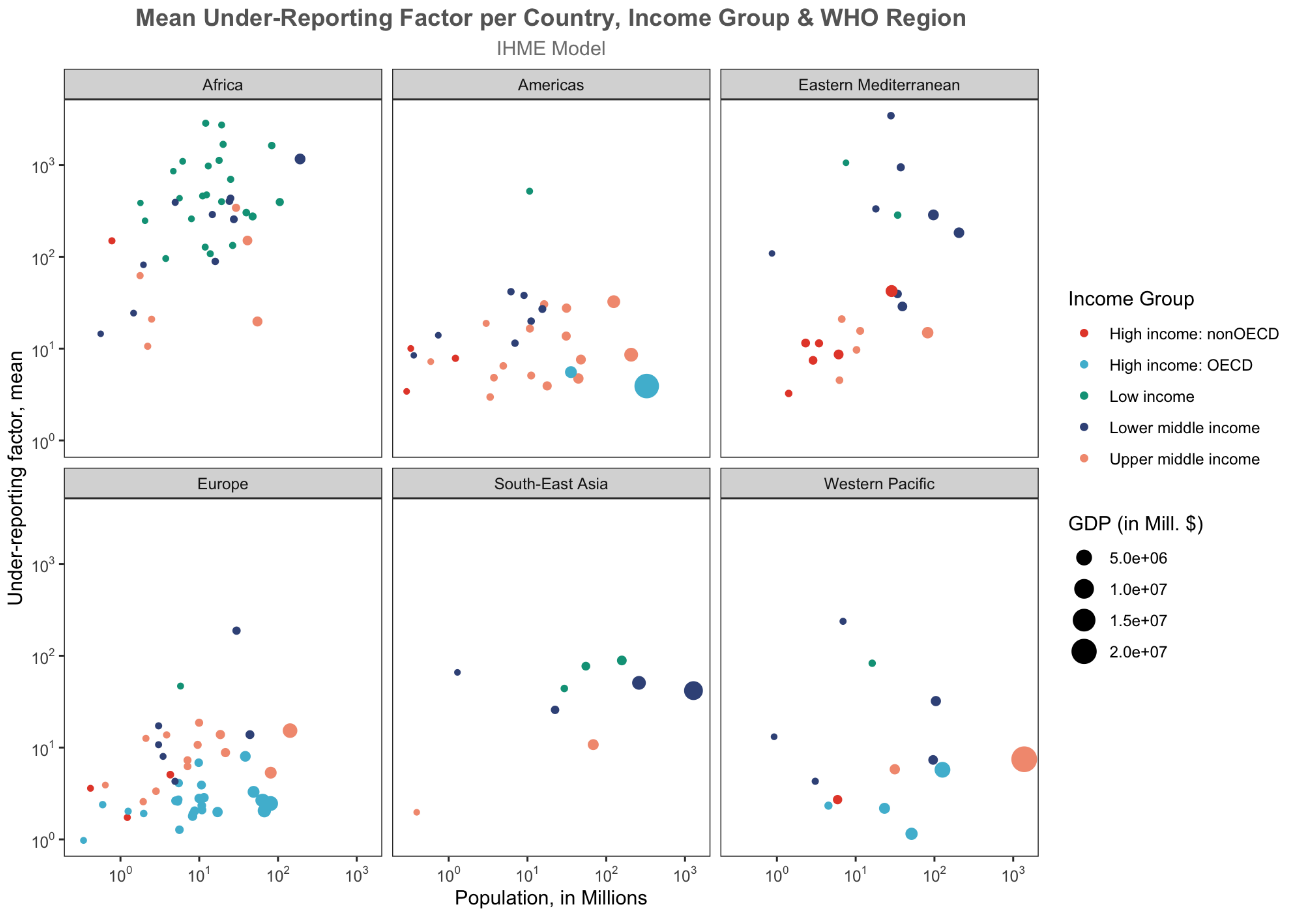

Fig. S11. Undercounting factors by WHO region and income group. Estimates of the factor accounting for missing confirmed cases: values larger than 1 indicate that a country is counting and confirming less COVID-19 cases than the real number. The reference period is the first semester of 2022. See the text for further details.

Prediction error

For each lineage we evaluate different scenarios with a) low and high values of underreporting in both source and target country b) three different basic reproduction numbers R_t that correspond to the range of growth rate values estimated from the phylogenetic reconstruction.

We infer from data the number of infected individuals with the emerging lineage in the target country m and we evaluate the prediction error as zero if this estimated number is included in the range identified by different epidemic scenarios. If the number of infected people evaluated from data is out of the range spanned by the epidemic curves, then the prediction error is evaluated as the root-mean-square error, normalized to the range of the data observed in the target country m, between observed and the closest simulated epidemic curve:

$$nRMSE(m) = \frac{1}{\max_t\left(I_m^{(data)}\right) - \min_t\left(I_m^{(data)}\right)}\sqrt{\frac{1}{n_t}\sum_{t=1}^{n_t}\left[I_m^{(data)}(t) - I_m^{(model)}(t)\right]^2} \tag{S27}$$

where n_t is the number of weeks with number of sequences greater than zero for the selected lineage in the considered country m, that is n_t is the number of available data points with not null infected people. Since the scenario simulations stop at the 3 week after sequencing was reported in country m, n_t is always $n_t = 2$. The idea behind the normalization by the data range is that it reflects the noise of reported sequences, i.e. if the sequencing rate is low, we expect a large variation and the sequencing data is less reliable. Prediction errors evaluated for all the considered lineages are shown in Figure 3 of the main document. All the panels report the nRMSE in each country as a function of both the number of daily passengers normalized to the total country population (x-axis, values for 100000 individuals) and the number of total collected daily sequences normalized to the total number of confirmed cases (y-axis, values for 100000 cases). Insets show the evaluated error in each country. Results assess that, in most of the country, the simulated scenarios encompass the data and the prediction error is evaluated as zero. Moreover, error values greater than zero can be found for countries with higher passenger flows.

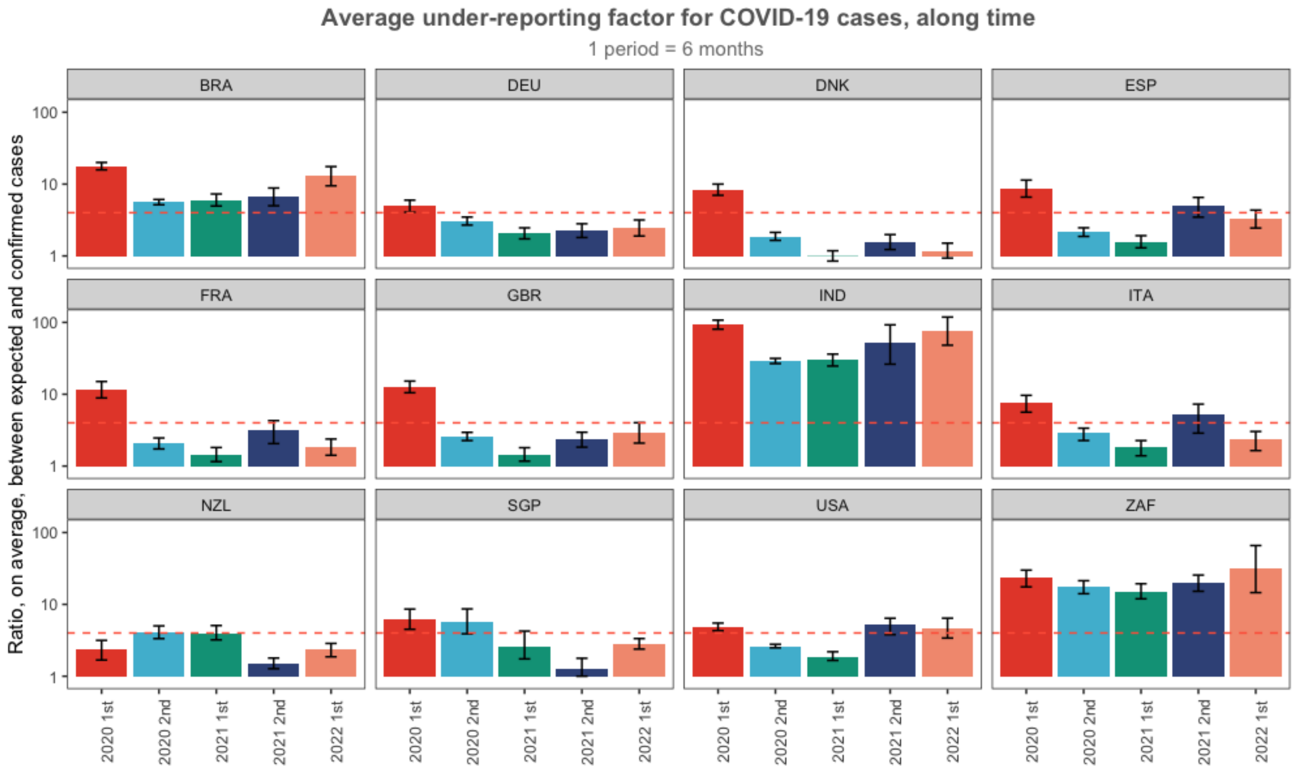

Fig. S12. Undercounting factors over time. Estimates of the factor accounting for missing confirmed cases as in Fig. S11, where each panel describes the evolution along periods of 6 months for some representative countries. The dashed line indicates the value 4. See the text for further details.

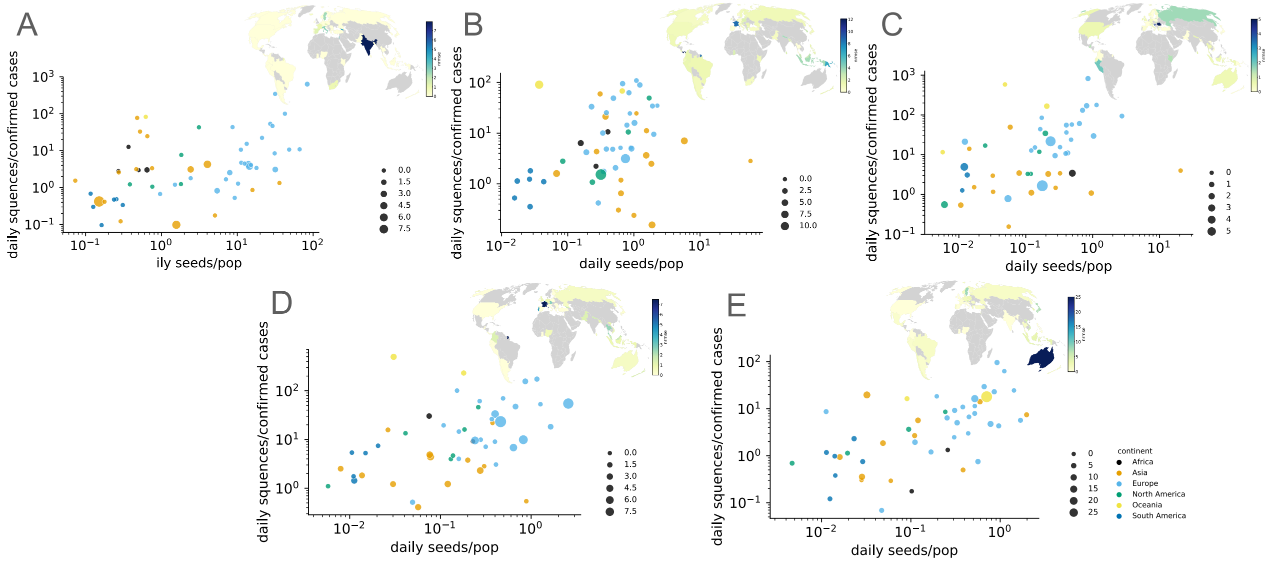

Fig. S13. Epidemic prediction errors. Estimated errors between the number of individuals infected with an emerging lineage and the epidemic curves simulated in the considered scenarios. X-axis show the number of daily passengers normalized to the population in each country (for 100, 000 individuals), y-axis report the number of collected daily sequences, without any classification per lineage, normalized to the total number of confirmed cases (for 100, 000 cases). Inset panels show the map of prediction errors in each country. Panels A-E refer to, respectively, Alpha, Delta, BA.1, BA.2 and BA.5 lineages.

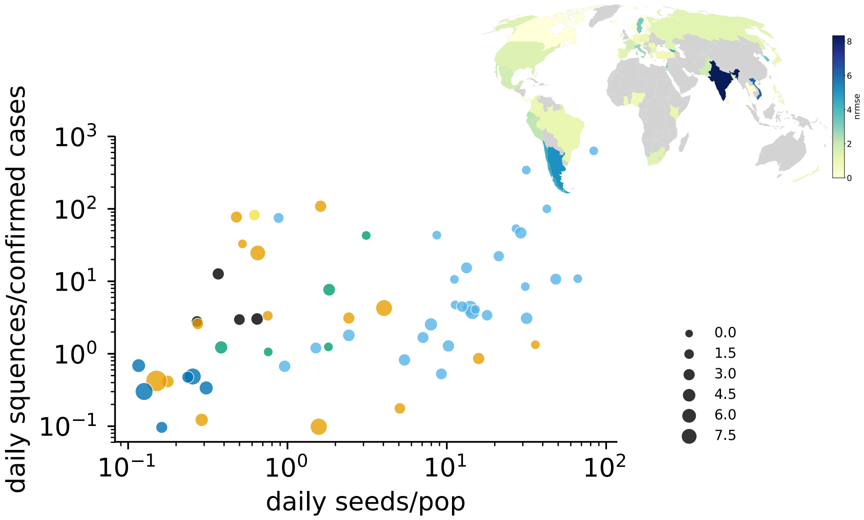

Fig. S14. Epidemic prediction errors with SIR model, Alpha lineage. Estimated errors between the number of individuals infected with an emerging lineage and the epidemic curves simulated in the considered scenarios. X-axis show the number of daily passengers normalized to the population in each country (for 100,000 individuals), y-axis report the number of collected daily sequences, without any classification per lineage, normalized to the total number of confirmed cases (for 100,000 cases). Inset panels show the map of prediction errors in each country.

Pandemic delay

The pandemic delay estimates the time needed since tMRCA for a specific variant to reach a certain percentage y in a target country. It depends in general on a large variety of factors as the reproduction number, the fraction of vaccinated, the variant's immune escape, season, weather conditions, the number, duration and strength of active non-pharmaceutical interventions (NPI), the national and international mobility and the epidemic situation. In the following estimation of the pandemic delay, we assume that the main driver/predictors for the pandemic risk are the international mobility, the effective reproduction number and the country specific epidemic situation.

We will use a simple framework to combine the measures that is based on the replicator equation [27], stating that the fraction of a new variant can be described by a simple logistic growth equation (illustrated for Delta lineage in Figure S15). It assumes that there are 2 competing populations, the mixed population of all preexisting variants of size N_{pre} and the population of the emergent variant N_x. According to the replicator equation, the evolution of the fraction x of the new variant in the whole population corresponds to

$$\frac{dx}{dt} = x(f - \tilde{f}) \tag{S28}$$

with f as the fitness of the new variant x and $\tilde{f}$ as the mean fitness, i.e.

$$\begin{aligned}\tilde{f} &= xf + (1-x)f_v \\ &= x(f - f_v) + f_v \ .\end{aligned} \tag{S29}$$

We can therefore rewrite the time-evolution to

$$\begin{aligned}\frac{dx}{dt} &= x(f - \tilde{f}) \\ &= x(f - [x(f - f_v) + f_v]) \\ &= x([f - f_v] - x[f - f_v]) \\ &= \Delta f(x - x^2)\end{aligned} \tag{S30}$$

that has the logistic function as general solution

$$x(t) = \frac{1}{1 + e^{-\Delta f t}c} = \frac{1}{1 + [1/x_0 - 1]\, e^{-\Delta f t}} \ , \tag{S31}$$

with x_0 as initial condition being the imported infected cases from the country of origin n_0 to the target country m

$$x(t_0, m|n_0) = x_0 = \frac{U_r(t_0, n_0) I_x(t_0, n_0)}{U_r(t_0, m) I_v(t_0, m)} \cdot \frac{F_{n_0}}{N_{n_0}} \cdot p_\infty(m|n_0) \ , \tag{S32}$$

with $t_0 = \text{tMRCA}$, $U_r(t_0, m)$ as the underreporting factor of cases in country m (introduced in Sec. III.2), $\frac{F_{n_0}}{N_{n_0}}$ as the probability of leaving the country via the WAN and $p_\infty(m|n_0)$ as the import risk (see Sec. II). Note that with Eqs. S31, S32 we assume that the initial import x_0 dominates, i.e. imports at later times can be neglected (otherwise a constant flux needs to be implemented). The fitness difference between the new variant vs. the already existing variant mix is approximated by

$$\Delta f = \ln R - \ln\left(R_{pre} = 1\right) = \ln R \tag{S33}$$

i.e. we assume that the reproduction number of the preexisting variant mix is one, motivated by the observed fluctuations around $R_{pre} = 1$ due to the behavioral and/or medical adaptation to the local epidemic situation.

The pandemic delay t_y is the time needed for the new variant to reach the fraction y of the infected population, where $t_y(m)$ for a specific country m is (rearranging Eq. S31)

$$t_y(m) = -\frac{1}{\Delta f} \ln\left(\frac{1-y}{[1/x_0(m) - 1]y}\right) \ . \tag{S34}$$

We can further simplify the pandemic delay by assuming that the initial import is small

$$\begin{aligned}t_y(m) &= \frac{1}{\Delta f}\left(\ln\left(\frac{1-x_0}{x_0}\right) - \ln\left(\frac{1-y}{y}\right)\right) \\ &\propto \frac{1}{\Delta f}\left(\ln\left(\frac{1-x_0}{x_0}\right)\right) \\ &\propto -\frac{\ln x_0}{\Delta f} \ .\end{aligned} \tag{S35}$$

However, this simplification is merely meant as a help to ease understanding of the functional relations. In the manuscript, we use explicitly Eq. S34.

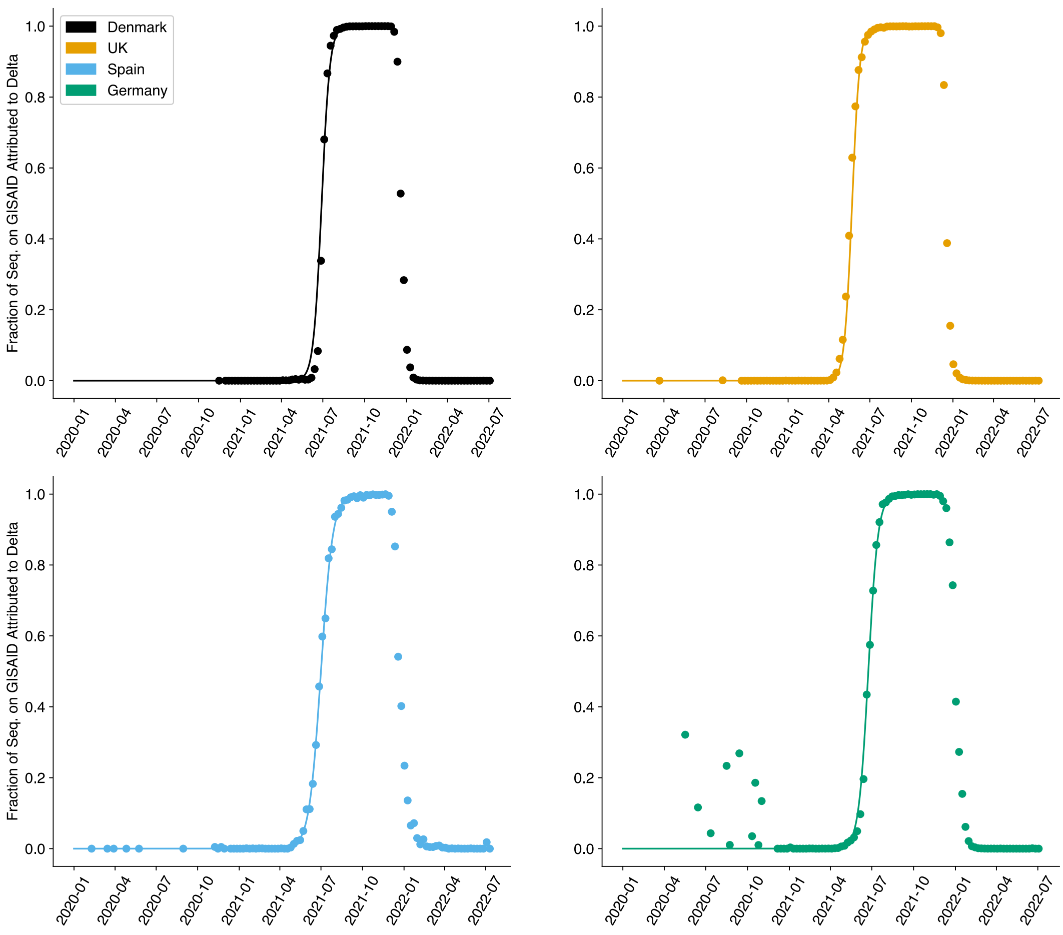

Fig. S15. The fraction of seq. on GISAID attributed to the Delta variant for four example countries. As described for the Alpha variant by Fort [27], the relative fraction of a new variant can be accurately described by a simple logistic growth equation (Eq. S31).

Information Distance

We also devise an alternative definition of distance on top of a network which embeds information from multiple-pathways diffusion as an additional comparison to the import risk measure. Distances based on the diffusive properties of the system have been of interest in recent years [10, 13]. Another key example is the Diffusion Distance [12] which estimates a metric distance between nodes based on how similarly the random walkers explore the network by using those nodes as sources, under the assumption that a mesoscale structure is recovered during the time scales in which the random walker explores its functional community.

Starting from Diffusion Distance definition, we propose an educated rewrite of the measure that fits the problem under study to predict arrival times of a random walker on the network, such as an infectious traveler from a source country. The probability $\mathbf{p}(t \mid i)$ of a walker to be in any point in the network at time t, starting from node i, embeds information of multiple paths via successive applications of the Laplacian operator. We introduce a new measure that merges this concept from Diffusion Distance and also embeds information from Effective Distance [10], namely, the idea that low probabilities $p_k(t \mid i)$ are associated with large distances. This can be embedded by taking the negative of the logarithm of the probability, in analogy with Shannon's entropy. We now introduce this candidate measure for diffusive dynamics which we define Information Distance:

$$D^{ID}_{(s \to k)}(t) = -\log_{10} p_k\left(t \mid s\right) \tag{S36}$$

in which $p_k\left(t \mid s\right)$ represents the $k-th$ entry associated with node k of the probability state $\mathbf{p}(t \mid s) = \mathbf{v_s} \cdot e^{-tL^{RW}}$. Here $\mathbf{v_s}$ is the initial condition probability for the walker starting from node s, the canonical vector with s-th component equal to 1. The random walk normalized Laplacian (L^{RW}) [28] term encodes the probability to move from node i to node j in its matrix elements. Its off-diagonal terms can be computed as the negative value of P_{ij}, which is directly estimated from the WAN weighted links as stated in subsection II.2. Given the multiple timescales involved in this definition, we evaluate the metric at different scales t to find the timescale at which $D^{ID}(t)$ performs better.

Lineage	Source	tMRCA	t_{50S}	Underrep. Fact.	Naive Seq. Rate [%]	Seq. Rate [%]
Alpha	GBR	13 Sep 2020	1 Nov 2020	2.6	0.19	0.075
Delta	IND	30 Aug 2020	7 Feb 2021	28.9	2.49	0.086
BA.1	ZAF	31 Oct 2021	5 Dec 2021	19.7	51.8	2.623
BA.2	ZAF	24 Oct 2021	19 Dec 2021	19.7	1.1	0.056
BA.5	ZAF	16 Jan 2022	17 Apr 2022	31.6	2.94	0.093
BA.2.75	IND	10 Apr 2022	12 Jun 2022	76.4	0.41	0.005

Table S1. Sequencing rates in the outbreak countries (Source) of SARS-CoV-2 B.1.1.7 (Alpha), B.1.617.2 (Delta), B.1.1.529 (BA.1), BA.2, BA.5 and BA.2.75 (Omicron) lineages. The outbreak countries (Source) are represented by their ISO alpha-3 codes (GBR: Great Britain, IND: India, ZAF: South Africa). The naive sequencing rate (Naive Seq. Rate) was computed by the ratio between new weekly cases (based on OWID-data [49]) and the weekly collected sequenced samples (based on GISAID-data [20]). We compute the final sequencing rate (Seq. Rate) by dividing through the underreporting factor (Underrep. Fact.) whose estimation is described in Sec. III.2. Both estimates are averaged for the lineage respective time-period between the median time of the most recent common ancestor (tMRCA) and the time when the first 50 samples got collected (t_{50S}).

References

1. K. Katoh and D. M. Standley, "MAFFT Multiple Sequence Alignment Software Version 7: Improvements in Performance and Usability," *Molecular Biology and Evolution*, vol. 30, pp. 772–780, 01 2013.
2. M. A. Suchard, P. Lemey, G. Baele, D. L. Ayres, A. J. Drummond, and A. Rambaut, "Bayesian phylogenetic and phylodynamic data integration using BEAST 1.10," *Virus Evolution*, vol. 4, 06 2018. vey016.
3. D. L. Ayres, M. P. Cummings, G. Baele, A. E. Darling, P. O. Lewis, D. L. Swofford, J. P. Huelsenbeck, P. Lemey, A. Rambaut, and M. A. Suchard, "BEAGLE 3: Improved Performance, Scaling, and Usability for a High-Performance Computing Library for Statistical Phylogenetics," *Systematic Biology*, vol. 68, pp. 1052–1061, 04 2019.
4. A. Rambaut, A. J. Drummond, D. Xie, G. Baele, and M. A. Suchard, "Posterior Summarization in Bayesian Phylogenetics Using Tracer 1.7," *Systematic Biology*, vol. 67, pp. 901–904, 04 2018.
5. J. Kennedy and R. Eberhart, "Particle swarm optimization," in *Proceedings of ICNN'95-international conference on neural networks*, vol. 4, pp. 1942–1948, IEEE, 1995.
6. H. Ritchie, E. Mathieu, L. Rodés-Guirao, C. Appel, C. Giattino, E. Ortiz-Ospina, J. Hasell, B. Macdonald, D. Beltekian, and M. Roser, "Coronavirus pandemic (covid-19)," *Our World in Data*, 2020. https://ourworldindata.org/coronavirus.
7. Cov-Lineages.org, "Global Lineage Reports," 2022.
8. Á. O'Toole, V. Hill, O. G. Pybus, A. Watts, I. I. Bogoch, K. Khan, J. P. Messina, H. Tegally, R. R. Lessells, J. Giandhari, S. Pillay, K. A. Tumedi, G. Nyepetsi, M. Kebabonye, M. Matsheka, M. Mine, S. Tokajian, H. Hassan, T. Salloum, G. Merhi, J. Koweyes, J. L. Geoghegan, J. de Ligt, X. Ren, M. Storey, N. E. Freed, C. Pattabiraman, P. Prasad, A. S. Desai, R. Vasanthapuram, T. F. Schulz, L. Steinbrück, T. Stadler, A. Parisi, A. Bianco, D. García de Viedma, S. Buenestado-Serrano, V. Borges, J. Isidro, S. Duarte, J. P. Gomes, N. S. Zuckerman, M. Mandelboim, O. Mor, T. Seemann, A. Arnott, J. Draper, M. Gall, W. Rawlinson, I. Deveson, S. Schlebusch, J. McMahon, L. Leong, C. K. Lim, M. Chironna, D. Loconsole, A. Bal, L. Josset, E. Holmes, K. St. George, E. Lasek-Nesselquist, R. S. Sikkema, B. Oude Munnink, M. Koopmans, M. Brytting, V. Sudha rani, S. Pavani, T. Smura, A. Heim, S. Kurkela, M. Umair, M. Salman, B. Bartolini, M. Rueca, C. Drosten, T. Wolff, O. Silander, D. Eggink, C. Reusken, H. Vennema, A. Park, C. Carrington, N. Sahadeo, M. Carr, G. Gonzalez, T. de Oliveira, N. Faria, A. Rambaut, and M. U. G. Kraemer, "Tracking the international spread of SARS-CoV-2 lineages B.1.1.7 and B.1.351/501Y-V2 with grinch," *Wellcome Open Research*, vol. 6, p. 121, sep 2021.
9. P. P. Klamser, A. Zachariae, B. F. Maier, O. Baranov, C. Jongen, F. Schlosser, and D. Brockmann, "Inferring country-specific import risk of diseases from the world air transportation network," *arXiv preprint*, 2023.
10. D. Brockmann and D. Helbing, "The Hidden Geometry of Complex, Network-Driven Contagion Phenomena," *Science*, vol. 342, pp. 1337–1342, dec 2013.
11. A. Gautreau, A. Barrat, and M. Barthélemy, "Global disease spread: Statistics and estimation of arrival times," *Journal of Theoretical Biology*, vol. 251, pp. 509–522, apr 2008.
12. M. De Domenico, "Diffusion Geometry Unravels the Emergence of Functional Clusters in Collective Phenomena," *Physical Review Letters*, vol. 118, p. 168301, apr 2017.
13. F. Iannelli, A. Koher, D. Brockmann, P. Hövel, and I. M. Sokolov, "Effective distances for epidemics spreading on complex networks," *Physical Review E*, vol. 95, p. 012313, jan 2017.
14. WHO, "WHO - Tracking SARS-CoV-2 variants," 2022.
15. A. Cori, N. M. Ferguson, C. Fraser, and S. Cauchemez, "A New Framework and Software to Estimate Time-Varying Reproduction Numbers During Epidemics," *American Journal of Epidemiology*, vol. 178, pp. 1505–1512, Nov. 2013.
16. L. Ferretti, C. Wymant, M. Kendall, L. Zhao, A. Nurtay, L. Abeler-Dörner, M. Parker, D. Bonsall, and C. Fraser, "Quantifying SARS-CoV-2 transmission suggests epidemic control with digital contact tracing," *Science (80-.).*, vol. 368, p. eabb6936, may 2020.
17. I. Dorigatti, L. Okell, A. Cori, N. Imai, M. Baguelin, S. Bhatia, A. Boonyasiri, Z. Cucunubá, G. Cuomo-Dannenburg, R. FitzJohn, H. Fu, K. Gaythorpe, A. Hamlet, W. Hinsley, N. Hong, M. Kwun, D. Laydon, G. Nedjati-Gilani, S. Riley, S. Van, E. Volz, H. Wang, R. Wang, C. Walters, X. Xi, C. Donnelly, A. Ghani, and N. Ferguson, "Report 4: Severity of 2019-novel coronavirus (nCoV)," p. 12, 2020.
18. M. Fabiani, M. Puopolo, C. Morciano, M. Spuri, S. Spila Alegiani, A. Filia, F. D\textquoterightAncona, M. Del Manso, F. Riccardo, M. Tallon, V. Proietti, C. Sacco, M. Massari, R. Da Cas, A. Mateo-Urdiales, A. Siddu, S. Battilomo, A. Bella, A. T. Palamara, P. Popoli, S. Brusaferro, G. Rezza, F. Menniti Ippolito, and P. Pezzotti, "Effectiveness of mRNA vaccines and waning of protection against SARS-CoV-2 infection and severe covid-19 during predominant circulation of the delta variant in Italy: retrospective cohort study," *BMJ*, vol. 376, 2022.
19. F. C. M. Kirsebom, N. Andrews, J. Stowe, S. Toffa, R. Sachdeva, E. Gallagher, N. Groves, A.-M. O'Connell, M. Chand, M. Ramsay, and J. L. Bernal, "COVID-19 vaccine effectiveness against the omicron (BA.2) variant in England," *Lancet Infect. Dis.*, vol. 22, pp. 931–933, jul 2022.
20. N. Andrews, J. Stowe, F. Kirsebom, S. Toffa, T. Rickeard, E. Gallagher, C. Gower, M. Kall, N. Groves, A.-M. O'Connell, D. Simons, P. B. Blomquist, A. Zaidi, S. Nash, N. Iwani Binti Abdul Aziz, S. Thelwall, G. Dabrera, R. Myers, G. Amirthalingam, S. Gharbia, J. C. Barrett, R. Elson, S. N. Ladhani, N. Ferguson, M. Zambon, C. N. J. Campbell, K. Brown, S. Hopkins, M. Chand, M. Ramsay, and J. Lopez Bernal, "Covid-19 Vaccine Effectiveness against the Omicron (B.1.1.529) Variant," *N. Engl. J. Med.*, vol. 386, no. 16, pp. 1532–1546, 2022.
21. Q. Wang, Y. Guo, S. Iketani, M. S. Nair, Z. Li, H. Mohri, M. Wang, J. Yu, A. D. Bowen, J. Y. Chang, J. G. Shah, N. Nguyen, Z. Chen, K. Meyers, M. T. Yin, M. E. Sobieszczyk, Z. Sheng, Y. Huang, L. Liu, and D. D. Ho, "Antibody evasion by SARS-CoV-2 Omicron subvariants BA.2.12.1, BA.4, & BA.5," *Nature*, 2022.

22. V. J. Hall, S. Foulkes, A. Charlett, A. Atti, E. J. M. Monk, R. Simmons, E. Wellington, M. J. Cole, A. Saei, B. Oguti, K. Munro, S. Wallace, P. D. Kirwan, M. Shrotri, A. Vusirikala, S. Rokadiya, M. Kall, M. Zambon, M. Ramsay, T. Brooks, C. S. Brown, M. A. Chand, S. Hopkins, N. Andrews, A. Atti, H. Aziz, T. Brooks, C. S. Brown, D. Camero, C. Carr, M. A. Chand, A. Charlett, H. Crawford, M. Cole, J. Conneely, S. D'Arcangelo, J. Ellis, S. Evans, S. Foulkes, N. Gillson, R. Gopal, L. Hall, V. J. Hall, P. Harrington, S. Hopkins, J. Hewson, K. Hoschler, D. Ironmonger, J. Islam, M. Kall, I. Karagiannis, O. Kay, J. Khawam, E. King, P. Kirwan, R. Kyffin, A. Lackenby, M. Lattimore, E. Linley, J. Lopez-Bernal, L. Mabey, R. McGregor, S. Miah, E. J. M. Monk, K. Munro, Z. Naheed, A. Nissr, A. M. O'Connell, B. Oguti, H. Okafor, S. Organ, J. Osbourne, A. Otter, M. Patel, S. Platt, D. Pople, K. Potts, M. Ramsay, J. Robotham, S. Rokadiya, C. Rowe, A. Saei, G. Sebbage, A. Semper, M. Shrotri, R. Simmons, A. Soriano, P. Staves, S. Taylor, A. Taylor, A. Tengbe, S. Tonge, A. Vusirikala, S. Wallace, E. Wellington, M. Zambon, D. Corrigan, M. Sartaj, L. Cromey, S. Campbell, K. Braithwaite, L. Price, L. Haahr, S. Stewart, E. D. Lacey, L. Partridge, G. Stevens, Y. Ellis, H. Hodgson, C. Norman, B. Larru, S. Mcwilliam, S. Winchester, P. Cieciwa, A. Pai, C. Loughrey, A. Watt, F. Adair, A. Hawkins, A. Grant, R. Temple-Purcell, J. Howard, N. Slawson, C. Subudhi, S. Davies, A. Bexley, R. Penn, N. Wong, G. Boyd, A. Rajgopal, A. Arenas-Pinto, R. Matthews, A. Whileman, R. Laugharne, J. Ledger, T. Barnes, C. Jones, D. Botes, N. Chitalia, S. Akhtar, G. Harrison, S. Horne, N. Walker, K. Agwuh, V. Maxwell, J. Graves, S. Williams, A. O'Kelly, P. Ridley, A. Cowley, H. Johnstone, P. Swift, J. Democratis, M. Meda, C. Callens, S. Beazer, S. Hams, V. Irvine, B. Chandrasekaran, C. Forsyth, J. Radmore, C. Thomas, K. Brown, S. Roberts, P. Burns, K. Gajee, T. M. Byrne, F. Sanderson, S. Knight, E. Macnaughton, B. J. L. Burton, H. Smith, R. Chaudhuri, K. Hollinshead, R. J. Shorten, A. Swan, R. J. Shorten, C. Favager, J. Murira, S. Baillon, S. Hamer, K. Gantert, J. Russell, D. Brennan, A. Dave, A. Chawla, F. Westell, D. Adeboyeku, P. Papineni, C. Pegg, M. Williams, S. Ahmad, S. Ingram, C. Gabriel, K. Pagget, P. Cieciwa, G. Maloney, J. Ashcroft, I. Del Rosario, R. Crosby-Nwaobi, C. Reeks, S. Fowler, L. Prentice, M. Spears, G. McKerron, K. McLelland-Brooks, J. Anderson, S. Donaldson, K. Templeton, L. Coke, N. Elumogo, J. Elliott, D. Padgett, M. Mirfenderesky, A. Cross, J. Price, S. Joyce, I. Sinanovic, M. Howard, T. Lewis, P. Cowling, D. Potoczna, S. Brand, L. Sheridan, B. Wadams, A. Lloyd, J. Mouland, J. Giles, G. Pottinger, H. Coles, M. Joseph, M. Lee, S. Orr, H. Chenoweth, C. Auckland, R. Lear, T. Mahungu, A. Rodger, K. Penny-Thomas, S. Pai, J. Zamikula, E. Smith, S. Stone, E. Boldock, D. Howcroft, C. Thompson, M. Aga, P. Domingos, S. Gormley, C. Kerrison, L. Marsh, S. Tazzyman, L. Allsop, S. Ambalkar, M. Beekes, S. Jose, J. Tomlinson, A. Jones, C. Price, J. Pepperell, M. Schultz, J. Day, A. Boulos, E. Defever, D. McCracken, K. Brown, K. Gray, A. Houston, T. Planche, R. Pritchard Jones, D. Wycherley, S. Bennett, J. Marrs, K. Nimako, B. Stewart, N. Kalakonda, S. Khanduri, A. Ashby, M. Holden, N. Mahabir, J. Harwood, B. Payne, K. Court, N. Staines, R. Longfellow, M. E. Green, L. E. Hughes, M. Halkes, P. Mercer, A. Roebuck, E. Wilson-Davies, L. Gallego, R. Lazarus, N. Aldridge, L. Berry, F. Game, T. Reynolds, C. Holmes, M. Wiselka, A. Higham, M. Booth, C. Duff, J. Alderton, H. Jory, E. Virgilio, T. Chin, M. Z. Qazzafi, A. M. Moody, R. Tilley, T. Donaghy, K. Shipman, R. Sierra, N. Jones, G. Mills, D. Harvey, Y. W. J. Huang, J. Birch, L. Robinson, S. Board, A. Broadley, C. Laven, N. Todd, D. W. Eyre, K. Jeffery, S. Dunachie, C. Duncan, P. Klenerman, L. Turtle, T. De Silva, H. Baxendale, and J. L. Heeney, "SARS-CoV-2 infection rates of antibody-positive compared with antibody-negative health-care workers in England: a large, multicentre, prospective cohort study (SIREN)," *Lancet*, vol. 397, pp. 1459–1469, apr 2021.
23. H. N. Altarawneh, H. Chemaitelly, M. R. Hasan, H. H. Ayoub, S. Qassim, S. AlMukdad, P. Coyle, H. M. Yassine, H. A. Al-Khatib, F. M. Benslimane, Z. Al-Kanaani, E. Al-Kuwari, A. Jeremijenko, A. H. Kaleeckal, A. N. Latif, R. M. Shaik, H. F. Abdul-Rahim, G. K. Nasrallah, M. G. Al-Kuwari, A. A. Butt, H. E. Al-Romaihi, M. H. Al-Thani, A. Al-Khal, R. Bertollini, P. Tang, and L. J. Abu-Raddad, "Protection against the Omicron Variant from Previous SARS-CoV-2 Infection," *N. Engl. J. Med.*, vol. 386, no. 13, pp. 1288–1290, 2022.
24. P. Stefanelli, F. Trentini, G. Guzzetta, V. Marziano, A. Mammone, M. Sane Schepisi, P. Poletti, C. Molina Grané, M. Manica, M. del Manso, X. Andrianou, M. Ajelli, G. Rezza, S. Brusaferro, S. Merler, and C.-. N. M. S. S. Group, "Co-circulation of SARS-CoV-2 Alpha and Gamma variants in Italy, February and March 2021," *Eurosurveillance*, vol. 27, no. 5, 2022.
25. L. Ferretti, A. Ledda, C. Wymant, L. Zhao, V. Ledda, L. Abeler-Dörner, M. Kendall, A. Nurtay, H.-Y. Cheng, T.-C. Ng, H.-H. Lin, R. Hinch, J. Masel, A. M. Kilpatrick, and C. Fraser, "The timing of COVID-19 transmission," *medRxiv*, 2020.
26. W. D. Green, N. M. Ferguson, and A. Cori, "Inferring the reproduction number using the renewal equation in heterogeneous epidemics," *Journal of The Royal Society Interface*, vol. 19, mar 2022.
27. H. Fort, "A very simple model to account for the rapid rise of the alpha variant of sars-cov-2 in several countries and the world," *Virus Research*, vol. 304, p. 198531, 2021.
28. M. Newman, "Networks: An Introduction," p. 800, 2018.
